# Growth parameters, phytochemicals, and antitumor activity of wild and cultivated ice plants (*Mesembryanthemum crystallinum* L.)

**DOI:** 10.1002/fsn3.4286

**Published:** 2024-06-21

**Authors:** Miguel Ángel Rincón‐Cervera, Tatiana Pagan Loeiro da Cunha‐Chiamolera, Tarik Chileh‐Chelh, Minerva Carmona‐Fernández, Miguel Urrestarazu, José Luis Guil‐Guerrero

**Affiliations:** ^1^ Food Technology Division, ceiA3, CIAMBITAL University of Almería Almería Spain; ^2^ Institute of Nutrition and Food Technology University of Chile Santiago Chile; ^3^ Vegetal Production Division University of Almería Almería Spain

**Keywords:** antioxidant activity, fatty acids, HT‐29 cells, *Mesembryanthemum crystallinum*, phytochemicals, soilless culture

## Abstract

The ice plant (*Mesembryanthemum crystallinum* L.) is a halophyte that could become an alternative crop because of its interest as a functional food and its adaptation to high‐saline soils. In this work, leaves from wild ice plants were compared with their cultivated counterparts in a soilless system at different salinities and light exposures for assessing growth parameters, moisture, fatty acid profiles, total carotenoids, phenolic compounds, vitamin C, antioxidant activity, and antiproliferative activity against the HT‐29 colorectal cancer cell line. Moisture ranged between 876 and 955 g kg^−1^, and wild plants contained higher proportions of α‐linolenic acid (58.7%–60.7% of total fatty acids) than cultivated ones (20.4%–36.6%). Vitamin C ranged between 819 and 1143 mg kg^−1^ fresh leaves. Higher salinity led to a larger production of carotenoids, whereas plant mass, total phenolic content, and antioxidant activity increased in plants grown using L8 NS1 and L8 AP67 lamps in comparison with white‐light ones. Phenolic profiles were assessed by LC coupled to a hybrid mass spectrometer Q‐Orbitrap. Total phenolic acid content was 3–4‐fold higher than that of flavonoids, and sinapic, *p*‐coumaric, gallic, 4‐hydroxybenzoic, and 2‐hydroxy‐4‐methoxybenzoic acids, as well as gallocatechin, occurred in all samples. Hydroalcoholic extracts of ice plant leaves showed dose‐ and time‐dependent antiproliferative activity against the HT‐29 human colorectal cancer cell line, and GI_50_ was between 920 and 977 μg mL^−1^ of plant extract. This work contributes to improving knowledge about the growth parameters, phytochemical profiles, and biological activities of wild and cultivated ice plants.

## Graphical abstract

Growth, phytochemicals, and antitumor activity of the ice plant were assessed. α‐Linolenic acid (18:3*n*‐3) was significantly higher in wild than in cultivated plants. Higher salinity levels led to increased total carotenoids in cultivated plants. Selected light treatments increased phenolic content and fresh plant mass. Leaf extracts showed antiproliferative effects against HT‐29 human colorectal cancer cells.

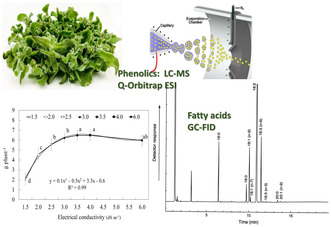

## 1 INTRODUCTION

The consumption of wild edible plants constitutes a growing trend in Western countries. They are often rich in phytochemicals, minerals, and vitamins that contribute to overall health. For instance, many wild greens contain higher levels of vitamins A and C compared to domesticated vegetables. These plants can offer dietary diversity, promoting a balanced and nutrient‐rich diet. Moreover, in the context of food security and sustainability, wild edible plants represent a valuable resource. Their resilience and adaptability to various environmental conditions make them a reliable food source that requires minimal human intervention (Bharucha & Pretty, 2010). Ice plant (*Mesembryanthemum crystallinum* L.) is an annual succulent wild edible vegetable native to South and East Africa that is currently distributed in several Mediterranean‐climate areas in Australia, the United States, Mexico, Chile, and European coastal areas (Rodríguez‐Hernández & Garmendia, 2022). This halophyte can grow in conditions of drought, high salinity, and high temperature, which makes this plant a suitable candidate to be grown in high saline soils not usable for conventional crops (Zhang et al., 2021).

Ice plant leaves (IPLs) are becoming a highly appreciated food ingredient because of their crispy, salty, and slightly sour flavor (Romojaro et al., 2013). Usually, IPLs are eaten raw in salads, used to enhance the flavor of seafood, or cooked (Loconsole et al., 2019). Besides their gastronomic value, IPLs have cosmetic and nutritional interest as sources of bioactive compounds (El‐Amier et al., 2021; Romojaro et al., 2013). IPLs are used as a moisturizing agent and to delay skin aging in cosmetic formulations and have further applications in folk medicine against eye and mouth infections, to treat fungal and bacterial diseases, diarrhea, and other disorders such as diabetes (Loconsole et al., 2019). It is therefore important to gain a deeper knowledge of the growth behavior and the phytochemical profile of this halophyte.

Biosynthesis and accumulation of phytochemicals relies on the soil and climate conditions to which the plant is subjected during growth. Nutrients and bioactive compounds in halophytes and other plants are generally dependent on biotic and abiotic stresses such as salinity and light radiation (Lima et al., 2021). In geographical locations such as the coastal areas of Southern Spain, wild specimens of IP are generally exposed to environmental stress conditions (drought, high temperatures, highly saline soils), which is related to higher concentrations of bioactive molecules. Controlled cultivation in growth chambers was developed to provide advantages such as increased productivity, product uniformity, and food security compared to the collection of wild specimens for human consumption (Lima et al., 2021).

Light exposure and its spectrum are important factors for plant development and metabolism (Nájera & Urrestarazu, 2019). LED lamps provide long‐life performance, low heat release, low cost, and irradiation with specific wavelengths in comparison with other radiation sources. Red light generally stimulates leaf area, whereas blue light increases total phenolic content (TPC) and antioxidant capacity (Kim et al., 2021). However, the influence of the type of light on the carotenoid content of IPL has been less explored, and the results are not conclusive (Zhang et al., 2021).

The antiproliferative activity of IPL extracts was assayed against several cancer cell lines, and both the extract concentration and the extracting solvent influenced this activity (Essa & Elsebaie, 2018; Rodrigues et al., 2014; Seo & Ju, 2019). In this sense, it is relevant to provide novel data regarding the role of such parameters on the anticancer activity of IPL extracts against different cancer cell lines.

The aim of this work was to assess the influence of IP type (wild vs. cultivated) on fatty acid (FA) profiles, TPC and Total Flavonoids Content (TFC), phenolic compound profiles, total carotenoids, and vitamin C. Growth parameters (different light and salinity treatments) were assayed for cultivated plants. Furthermore, the antioxidant activity and antiproliferative actions of IPL extracts against HT‐29 human colorectal cancer cells were studied.

## 2 METHODS AND MATERIALS

### 2.1 Solvents and reagents

Unless otherwise stated, all solvents and reagents used in the current work were purchased from Merck (Madrid, Spain).

### 2.2 Samples and growth conditions

Information regarding *M. crystallinum* plants studied in this work is shown in Table 1. IPLs were collected in the locations listed in Table 1, whereas cultivated plants were grown in a soilless system with LED lamps at the University of Almería in controlled growth chambers (10 × 2.5 m) between May and July 2022. Growth, fertigation, and lighting conditions applied to cultivated plants are detailed in File S1. Two independent experiments were performed. Experiment 1 used three nutrient solutions with different electrical conductivity (EC): 3.0 (C1), 4.0 (C2), and 6.0 (C3) dS m^−1^. A Roblan® L18 T8 white LED lamp (Toledo, Spain) was used in the assays. The composition of the nutrient solutions is shown in Table S1. In Experiment 2, four LED lamps were evaluated (treatments L1 to L4) (Table 1). The spectrum profile of each LED lamp is shown in Figure S1. The EC of the nutrient solution used was 3.0 dS m^−1^. The pH of the nutrient solutions in both trials was maintained at 5.8 with the addition of nitric acid. Once in the laboratory, samples were labeled, weighed, measured, and stored at −70°C until analysis. IPL (2 g) were placed in a forced air oven at 105°C until a constant weight was reached to determine the moisture content.

**TABLE 1:** Data on status, harvesting location, and cultivation variables of *M. crystallinum* samples used in this work.

Sample code	Status	Location	Conductivity of the nutrient solution/soil of collection (dS m^−1^)	Lamp type
WT	Wild	El Toyo, Almería (36.837729, −2.327154)	13.2	‐
WU	Wild	University Campus, Almería (36.831494, −2.401189)	16.4	‐
C1	Cultivated	Growth chamber, University of Almería	3.0	L18 T8 Roblan®
C2	Cultivated	Growth chamber, University of Almería	4.0	L18 T8 Roblan®
C3	Cultivated	Growth chamber, University of Almería	6.0	L18 T8 Roblan®
L1	Cultivated	Growth chamber, University of Almería	3.0	L18 T8 Roblan®
L2	Cultivated	Growth chamber, University of Almería	3.0	L18 NS12 Valoya®
L3	Cultivated	Growth chamber, University of Almería	3.0	L18 NS1 Valoya®
L4	Cultivated	Growth chamber, University of Almería	3.0	L18 AP67 Valoya®

### 2.3 Fatty acids

The FA profiles of IPL were obtained after direct derivatization to FA methyl esters (FAME) and further analysis by gaschromatography coupled with a flame ionization detector (GC‐FID) as previously described (Lyashenko et al., 2019). This procedure is fully detailed in File S1.

### 2.4 Total phenolics and flavonoids content

The extraction of phenolic compounds was carried out according to a previous work and is detailed in File S1 (Lyashenko et al., 2021). TPC was measured using the Folin–Ciocalteu (F–C) assay according to Singleton et al. (1999) with minor modifications. Results were reported as mg of gallic acid equivalents (GAE) per kg of fresh weight (fw) using a standard curve of gallic acid for quantification.

The total flavonoid content (TFC) of the phenolic extract was determined according to Zou et al. (2004) with some modifications (File S1). Results were reported as mg of quercetin equivalents (QE) per kg fw using a standard curve of quercetin.

### 2.5 Phenolic compound profiles

Phenols were identified using liquid chromatography coupled to a hybrid mass spectrometer, Q‐Orbitrap Thermo Fisher Scientific, using electrospray ionization (ESI) in positive and negative ion modes according to the procedure described in File S1. Data on LC–MS parameters used for the analysis of phenolic‐enriched extracts of IPL are detailed in Table S2.

### 2.6 Total carotenoids

Carotenoids were extracted with diethyl ether after saponification, according to the procedure detailed in File S1. After solvent evaporation, the residue was dissolved in acetone, and the absorbance was measured at 444 nm. Total carotenoids were quantified using a calibration curve made with pure β‐carotene in acetone, and the results were reported as mg carotenoids kg^−1^ fw.

### 2.7 Vitamin C

Extraction and quantification of vitamin C were carried out according to Volden et al. (2009) with minor modifications (File S1). Fresh leaves were minced and extracted with an aqueous solution of oxalic acid (1% w/v), and vitamin C (ascorbic acid plus dehydroascorbic acid) was measured by reverse‐phase HPLC (RP‐HPLC) at 254 nm using an external calibration. Results were recorded as mg kg^−1^ fw.

### 2.8 Antioxidant activity

ABTS and DPPH methods were selected to assess the antioxidant activity of sample extracts. The extraction was carried out according to previous work with some modifications (File S1) (Forbes‐Hernández et al., 2017). The values of ABTS and DPPH were reported as mg of Trolox equivalents kg^−1^ dw (mg TE kg^−1^ dw).

### 2.9 Antiproliferative activity of IPL extracts on HT‐29 and CCD‐18 cell lines

The antiproliferative activity of hydroalcoholic extracts (methanol:water 60:40 v/v) of IPL against HT‐29 human colon cancer cells and CCD‐18 normal human fibroblast colonic cells was checked using the MTT assay as described by Lyashenko et al. (2021) (File S1).

### 2.10 Statistical analysis

Analyses were carried out in triplicate, and results were reported as mean value ± standard deviation. Data were assessed for normality using a Shapiro–Wilk test and submitted to a one‐way ANOVA, and the comparison of means was made using the Duncan's multiple range test. The linear correlations among all parameters were assessed at *p* < .05 and *p* < .001. Statistical analyses were performed using Statgraphics© Centurion XVI (StatPoint Technologies, Warrenton‐Virginia, USA).

## 3 RESULTS

### 3.1 Effect of fertigation and light on growth parameters in cultivated *M. crystallinum*

#### 3.1.1 Fertigation parameters

In Experiment 1, the EC value remained above the supplied fertigation value (Figure S2A). The EC for the C1 treatment showed a slight decrease, while treatments C2 and C3 showed steady increases as the plants grew. The mean of the C1 treatment was 80% lower than the other treatments (Figure S2B). Drainage pH ranged from 0.8 points above the fertigation in the first week of cultivation to values below the input in the last week of crop (Figure S2C), with no significant differences in the mean values throughout the crop (Figure S2D). In Experiment 2, there were no statistical differences in EC over the weeks (Figure S3A). However, the mean EC of the treatments with agricultural LED lights (L2, L3, and L4) was 18.8% higher compared with the control (L1) (Figure S3B). There were no statistical differences in the pH values of the drains, with a mean of 6.6 (Figure S3C,D).

#### 3.1.2 Fertigation uptake and growth parameters

**FIGURE 1:**
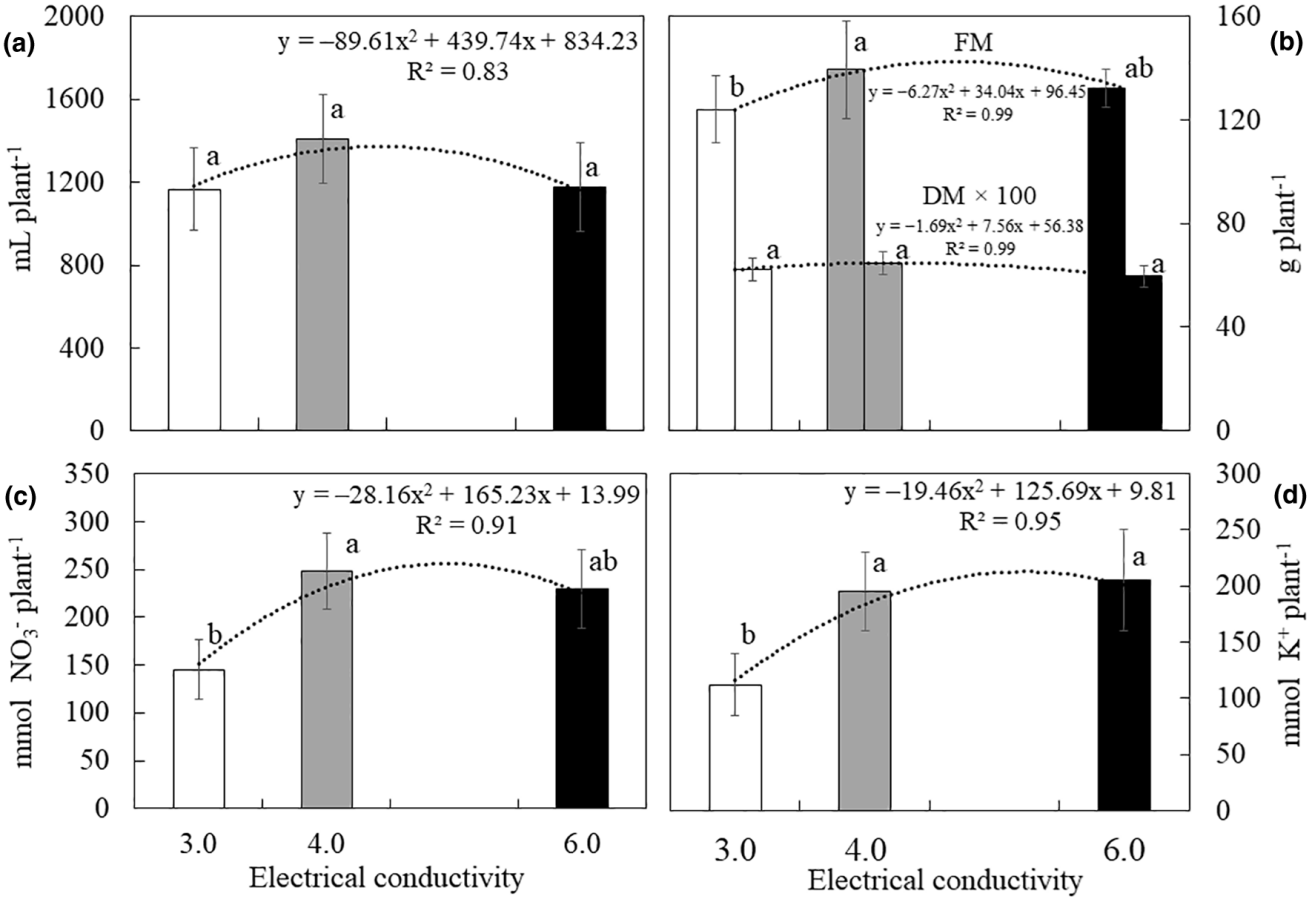
Uptake of water (a), fresh (FM) and dry mass (DM) (b), nitrate (c), and potassium (d) versus different electrical conductivity of the nutrient solutions (dS m^−1^) for *M. crystallinum* soilless culture. Different letters indicate significant differences for each parameter (*p* < .05) according to Tukey's test.

There was no statistical difference in fertigation uptake or plant dry mass in Experiment 1 (Figure 1a,b respectively). There was a statistical difference for the fresh mass of plants. The C2 treatment (138 g plant^−1^) showed a significant increase of 10% compared to C1 (124 g plant^−1^) (*p* < .05). However, there was no statistical difference for dry mass. It was also observed that there was a statistical difference in the uptake of nitrate and potassium (Figure 1c,d, respectively), with the highest values in C2 and C3 plants.

**FIGURE 2:**
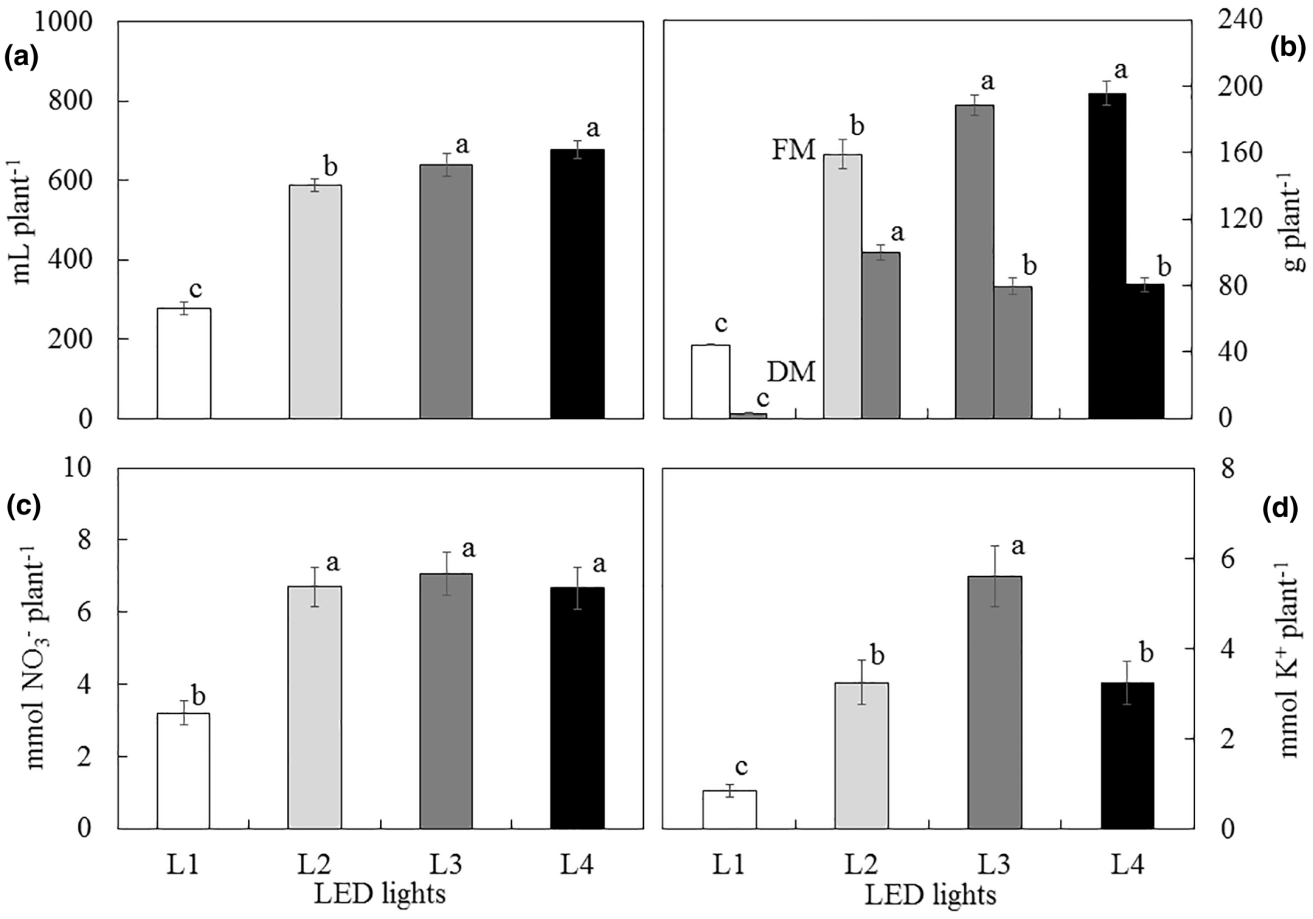
Uptake of water (a), fresh (FM) and dry mass (DM) (b), nitrate (c), and potassium (d) versus spectrum of illumination system for *M. crystallinum* soilless culture L1: T8 Roblan®; L2: NS12 Valoya®; L3: L18 NS1 Valoya®; L4: L18 AP67 Valoya®. Different letters indicate significant differences for each parameter (*p* < .05) according to Tukey's test.

In Experiment 2, L2, L3, and L4 plants recorded higher values of fertigation uptake compared to the control treatment (L1) (Figure 2a). Concerning the fresh mass of the plants, C2 (138 g plant^−1^) showed a significant increase of 10% compared to C1 (124 g plant^−1^) (*p* < .05); however, there was no statistical difference for dry mass. In the illumination experiment as well as in the fertigation absorption, specimens grown with lamps manufactured for horticulture (L2 to L4) showed a significant increase in fresh and dry mass compared with the control treatment (L1) (Figure 2b). These trends have been previously described (Haddaji et al., 2023). Finally, nitrate and potassium absorption had a similar trend to the values of fertigation uptake versus the light spectra (Figure 2c,d), and the lowest absorption values of nitrate and potassium were found in the control treatment (L1).

### 3.2 Fatty acids

FA profiles of IPL are shown in Table 2. Wild and cultivated specimens showed significant differences regarding saturated FA (SFA), monounsaturated FA (MUFA), and polyunsaturated FA (PUFA). Palmitic acid (PA, 16:0) was the most abundant SFA in all cases (16.6%–17.4% of total FA in wild plants and 31.8%–41.1% in cultivated plants). Oleic acid (OA, 18:1*n*‐9) was the predominant MUFA (1.9%–2.9% in wild plants and 7.7%–10.0% in cultivated plants). Regarding PUFA, the most significant difference was found for α‐linolenic acid (ALA, 18:3*n*‐3) (58.7%–60.7% for wild plants and 20.4%–36.6% for cultivated plants). Within wild samples, no significant differences were found for PA, OA, and ALA. Within cultivated plants, salinity differences had no significant effect on PA, OA, and ALA. However, light treatment led to differences in FA profiles; L4 and L3 plants significantly induced the highest proportions of PA (41.1% and 40.6%) and the lowest proportions of ALA (20.4% and 23.2%).

**TABLE 2:** Fatty acid profiles (FA% of total FA) of leaves from wild and cultivated *M. crystallinum* plants.

Fatty acids	Wild		Cultivated						
	WT	WU	C1	C2	C3	L1	L2	L3	L4
12:0	n.d.	n.d.	0.1 ± 0.1^ab^	n.d.	n.d.	0.1 ± 0.0^ab^	0.1 ± 0.0^ab^	0.2 ± 0.0^a^	0.1 ± 0.0^ab^
13:0	0.9 ± 0.1^c^	0.9 ± 0.0^c^	1.4 ± 0.1^ab^	1.1 ± 0.3^bc^	1.3 ± 0.3^ab^	0.9 ± 0.1^c^	1.3 ± 0.0^ab^	1.3 ± 0.1^ab^	1.6 ± 0.1^a^
14:0	0.6 ± 0.1^cd^	0.9 ± 0.1^a^	0.8 ± 0.0^ab^	0.6 ± 0.1^bc^	0.5 ± 0.1^cd^	0.6 ± 0.1^cd^	0.4 ± 0.1^d^	0.6 ± 0.0^bc^	0.4 ± 0.0^cd^
15:0	0.2 ± 0.0^bc^	n.d.	0.4 ± 0.0^b^	0.3 ± 0.1^bc^	0.3 ± 0.1^bc^	0.5 ± 0.3^b^	0.3 ± 0.0^bc^	1.4 ± 0.2^a^	0.5 ± 0.1^b^
16:0	16.6 ± 0.3^d^	17.4 ± 0.4^d^	31.9 ± 1.6^c^	32.2 ± 0.4^c^	31.8 ± 0.6^c^	32.7 ± 1.6^c^	35.8 ± 1.9^b^	40.6 ± 0.8^a^	41.1 ± 0.5^a^
18:0	0.7 ± 0.4^de^	n.d.	1.2 ± 0.6^cd^	1.0 ± 0.1^cd^	0.9 ± 0.1^d^	1.7 ± 0.1^bc^	2.0 ± 0.1^ab^	2.3 ± 0.3^ab^	2.4 ± 0.3^a^
20:0	0.4 ± 0.2^ab^	0.6 ± 0.3^ab^	n.d.	n.d.	n.d.	1.1 ± 0.2^a^	1.3 ± 0.4^a^	0.8 ± 1.1^ab^	0.6 ± 0.4^ab^
22:0	0.8 ± 0.1^cd^	0.9 ± 0.4^cd^	1.9 ± 0.0^ab^	1.8 ± 0.1^ab^	2.0 ± 0.1^ab^	1.4 ± 0.1^bc^	2.0 ± 0.4^a^	0.6 ± 0.3^d^	1.4 ± 0.4^bc^
∑SFA	20.0 ± 0.9^d^	20.7 ± 0.5^d^	37.7 ± 0.8^c^	37.0 ± 0.4^c^	36.8 ± 0.2^c^	38.8 ± 1.3^c^	44.2 ± 3.2^b^	47.8 ± 2.9^a^	48.0 ± 0.6^a^
14:1 *n*‐5	0.8 ± 0.0^c^	1.8 ± 0.3^abc^	1.6 ± 0.0^bc^	1.4 ± 0.2^bc^	1.8 ± 0.3^abc^	1.8 ± 0.2^abc^	2.3 ± 1.3^ab^	2.3 ± 0.1^ab^	2.7 ± 0.4^a^
16:1 *n*‐7	1.3 ± 0.1^ab^	n.d.	1.5 ± 0.1^a^	1.3 ± 0.1^ab^	1.4 ± 0.2^ab^	1.0 ± 0.3^bc^	0.8 ± 0.3^c^	0.9 ± 0.1^c^	0.6 ± 0.1^c^
18:1 *n*‐9	2.9 ± 0.1^c^	1.9 ± 0.8^c^	8.0 ± 0.1^b^	8.5 ± 0.1^b^	8.1 ± 0.1^b^	7.8 ± 0.7^b^	7.7 ± 0.8^b^	7.9 ± 0.2^b^	10.0 ± 0.7^a^
18:1 *n*‐7	0.2 ± 0.0^cde^	n.d.	0.4 ± 0.0^bcd^	0.3 ± 0.1^de^	0.2 ± 0.1^de^	0.3 ± 0.1^bcd^	0.8 ± 0.3^a^	0.5 ± 0.1^bc^	0.6 ± 0.1^ab^
∑MUFA	5.2 ± 0.1^c^	3.7 ± 1.1^d^	11.5 ± 0.1^b^	11.4 ± 0.6^b^	11.5 ± 0.8^b^	10.9 ± 0.8^b^	11.6 ± 0.5^b^	11.5 ± 0.4^b^	13.9 ± 0.2^a^
18:2 *n*‐6	16.2 ± 0.6^ab^	14.7 ± 0.6^c^	15.3 ± 0.4^bc^	15.4 ± 0.2^bc^	15.2 ± 0.1^bc^	17.1 ± 0.2^a^	15.9 ± 0.8^abc^	15.5 ± 1.0^bc^	15.0 ± 0.6^bc^
18:3 *n*‐3	58.7 ± 0.1^a^	60.7 ± 2.6^a^	35.7 ± 0.5^b^	36.3 ± 0.6^b^	36.6 ± 0.6^b^	31.6 ± 2.8^c^	27.3 ± 2.8^d^	23.2 ± 0.5^e^	20.4 ± 0.4^e^
18:4 *n*‐3	0.1 ± 0.1^c^	0.3 ± 0.4^c^	n.d.	n.d.	n.d.	1.7 ± 0.6^ab^	1.1 ± 0.4^bc^	2.1 ± 1.0^ab^	2.7 ± 0.8^a^
∑PUFA	75.0 ± 0.9^a^	75.6 ± 1.6^a^	51.0 ± 0.9^b^	51.6 ± 0.8^b^	51.7 ± 0.7^b^	50.3 ± 2.0^b^	44.3 ± 2.3^c^	40.8 ± 2.5^cd^	38.1 ± 1.1^d^
Total FA (g kg^−1^ dw)	35 ± 6^a^	36 ± 6^a^	29 ± 4^ab^	26 ± 7^ab^	25 ± 3^ab^	28 ± 4^ab^	27 ± 6^b^	31 ± 2^ab^	39 ± 3^a^

*Note*: Results are reported as mean value ± standard deviation (*n* = 3). Different superscript letters in a row means significant differences among values (*p* < .05).

Abbreviations: MUFA, monounsaturated FA; n.d., not detected; PUFA, polyunsaturated FA; SFA: saturated FA.

### 3.3 Moisture

The moisture of the wild samples was 944–945 g kg^−1^, and no significant differences were found among plants cultivated under different salinities (950–955 g kg^−1^) (Table 3). Moisture of plants cultivated at different light treatments showed significantly lower values, particularly L2 (876), L3 (899), and L4 (896 g kg^−1^) lamps.

**TABLE 3:** Moisture, total carotenoids, total phenolic content, total flavonoids, vitamin C contents, and antioxidant activity of leaves from wild and cultivated *M. crystallinum* plants.

Samples	Moisture (g kg^−1^ fw)	Total carotenoids (mg kg^−1^ fw)	TPC (mg GAE kg^−1^ fw)	TFC (mg QE kg^−1^ fw)	Vitamin C (mg kg^−1^ fw)	ABTS (mg TE kg^−1^ dw)	DPPH (mg TE kg^−1^ dw)
Wild							
WT	945 ± 4^a^	139 ± 3^b^	2190 ± 13^c^	639 ± 29^d^	1143 ± 33^a^	7816 ± 692^abc^	3084 ± 34^c^
WU	944 ± 3^a^	177 ± 4^a^	3378 ± 74^a^	981 ± 5^a^	1011 ± 64^b^	8641 ± 52^a^	4020 ± 137^a^
Cultivated							
Saline treatments							
C1	950 ± 4^a^	97 ± 4^c^	1707 ± 49^d^	464 ± 41^ef^	1018 ± 42^b^	7163 ± 479^bc^	3336 ± 336^bc^
C2	953 ± 4^a^	104 ± 1^c^	1584 ± 100^d^	281 ± 11^g^	872 ± 17^cd^	8584 ± 153^a^	3614 ± 116^abc^
C3	955 ± 2^a^	164 ± 19^a^	1775 ± 25^d^	526 ± 92^e^	998 ± 24^b^	8047 ± 756^ab^	3758 ± 331^ab^
Light treatments							
L1	918 ± 6^b^	65 ± 3^e^	1581 ± 138^d^	384 ± 2^f^	980 ± 47^b^	3686 ± 374^d^	1233 ± 195^d^
L2	876 ± 6^d^	76 ± 1^de^	1831 ± 20^d^	844 ± 25^bc^	974 ± 4^b^	6902 ± 569^c^	3405 ± 200^bc^
L3	899 ± 7^c^	88 ± 0^cd^	2304 ± 32^c^	762 ± 23^c^	819 ± 25^d^	8838 ± 461^a^	3894 ± 171^ab^
L4	896 ± 1^c^	86 ± 3^cd^	2643 ± 87^b^	864 ± 22^b^	956 ± 68^bc^	8299 ± 403^a^	3669 ± 257^ab^

*Note*: Results are reported as mean value ± standard deviation (*n* = 3). Different superscript letters in each column means significant differences among values (*p* < .05).

Abbreviations: TFC, total flavonoids content; TPC, total phenols content.

### 3.4 Total carotenoids

Carotenoid concentrations are reported in Table 3. The lowest values were found in cultivated plants under different light treatments (65–88 mg kg^−1^ fw). An increase in salinity led to increased total carotenoids (from 97 to 164 mg kg^−1^ in C1 and C3 plants), and leaves from wild plants showed significantly higher values than those of cultivated plants, except for C3 (164 mg kg^−1^ fw), which was comparable to WU (177 mg kg^−1^ fw) and even higher than total carotenoids from WT (139 mg kg^−1^ fw).

### 3.5 Total phenols and flavonoids

The highest TPC measured by the F‐C method was found in the leaves of WU and L4 plants (3378 and 2643 mg GAE kg^−1^ fw) (Table 3). WT and L3 plants showed intermediate values (2190 and 2304 mg GAE kg^−1^ fw), whereas the lowest concentrations were found in C1, C2, C3 (1584–1775 mg GAE kg^−1^ fw) and in L1 and L2 (1581 and 1831 mg GAE kg^−1^ fw), with no significant differences among them.

Regarding TFC, the highest concentrations were found in WU and L4 specimens (981 and 864 mg QE kg^−1^ fw), as observed also for TPC (Table 3). Leaves from L2 and L4 had similar TFC. WT and L3 plants showed intermediate values (639 and 762 mg QE kg^−1^ fw), whereas the lowest concentrations were found in the remaining cultivated plants (C1, C2, C3, and L1) (281–526 mg QE kg^−1^ fw).

### 3.6 Phenolic compounds profiles

Plant extracts with significantly higher TPC (>2 g GAE kg^−1^ fw), including WT, WU, L3 and L4, were subjected to a chromatographic analysis by HPLC–MS/MS to identify their main phenols. Phenolic acids were the main compounds identified in all analyzed extracts (Table 4): sinapic acid, *p*‐coumaric acid, gallic acid, 4‐hydroxybenzoic acid, and 2‐hydroxy‐4‐methoxybenzoic acid were found in all samples; coumaroylquinic acid isomer and DL‐*p*‐hydroxyphenyllactic acid were found only in cultivated plants; and caffeic acid occurred only in one of the wild specimens. Concerning flavonoids, a catechin derivative, i.e., gallocatechin, occurred in all samples, especially in L1‐L4, while epicatechin gallate (−) and the flavonoid quercetin were detected only in wild IPL.

**TABLE 4:** Phenolic compounds profiles (individual phenolic% of total phenolic area reported by the LC–MS system).

	WT	WU	C1	C2	C3	L1	L2	L3	L4
Coumaroylquinic acid isomer	n.d.	n.d.	8.5 ± 1.0^a^	2.1 ± 1.2^bc^	0.5 ± 0.9^d^	3.3 ± 1.1^b^	0.6 ± 1.1	8.9 ± 1.9^a^	0.9 ± 1.4^cd^
Gallocatechin (−)	4.4 ± 1.9^cd^	7.4 ± 2.8^c^	3.1 ± 1.3^d^	3.0 ± 1.6^d^	4.1 ± 2.0^cd^	28.8 ± 3.9^b^	57.8 ± 6.6^a^	47.4 ± 9.0^a^	59.6 ± 9.2^a^
DL*‐p*‐Hydroxyphenyllactic acid	n.d.	n.d.	23.1 ± 7.5^a^	17.3 ± 5.0^b^	n.d.	n.d.	n.d.	n.d.	n.d.
*p*‐coumaric acid glucoside	5.7 ± 2.3^b^	24.2 ± 7.8^a^	n.d.	n.d.	n.d.	3.3 ± 1.9^b^	n.d.	n.d.	n.d.
Caffeic acid	1.2 ± 1.7^a^	n.d.	n.d.	n.d.	n.d.	n.d.	n.d.	n.d.	n.d.
Sinapic acid	0.8 ± 1.4^e^	0.8 ± 1.8^e^	27.8 ± 7.9^b^	19.4 ± 6.0^bc^	57.6 ± 10.3^a^	4.3 ± 2.0^d^	9.3 ± 3.9^cd^	5.2 ± 2.7^d^	14.7 ± 6.8^c^
Epicatechin gallate (−)	0.5 ± 1.0^a^	0.4 ± 1.2^a^	n.d.	n.d.	n.d.	n.d.	0.8±	n.d.	n.d.
Quercetin	2.2 ± 2.9^a^	2.8 ± 3.2^a^	n.d.	n.d.	n.d.	n.d.	n.d.	n.d.	n.d.
*p*‐Coumaric acid	79.7 ± 8.9^a^	53.3 ± 10.2^b^	18.7 ± 5.9^c^	4.1 ± 2.3^e^	5.9 ± 2.9^de^	3.4 ± 1.8	11.7 ± 2.8^cd^	11.4 ± 4.5^cd^	2.9 ± 1.4^e^
Ferulic acid	1.4 ± 1.3^b^	6.0 ± 3.0^a^	1.1 ± 1.8^b^	n.d.	n.d.	n.d.	n.d.	n.d.	n.d.
Gallic acid	0.5 ± 1.8^c^	1.0 ± 1.6^bc^	5.2 ± 2.6^a^	1.3 ± 1.9^bc^	2.4 ± 1.6^b^	0.9 ± 1.8^c^	2.0 ± 1.9^b^	3.9 ± 2.7^ab^	1.5 ± 2.5^b^
4‐Hydroxybenzoic acid/salicylic acid (isomers)	0.4 ± 1.5^f^	1.8 ± 1.9^e^	3.1 ± 2.1^de^	0.9 ± 1.8^e^	0.6 ± 1.7^ef^	51.3 ± 7.3^a^	5.6 ± 2.0^cd^	14.1 ± 4.6^b^	10.4 ± 3.8^bc^
2‐Hydroxy‐4‐methoxybenzoic acid/vanillic acid (isomers)	3.2 ± 2.4^e^	2.3 ± 1.9^e^	9.4 ± 3.7^cd^	51.9 ± 9.9^a^	28.9 ± 6.7^b^	4.7 ± 3.1^de^	12.2 ± 4.4^c^	9.1 ± 3.8^c^	10.0 ± 3.6^c^

*Note*: Results are reported as mean value ± standard deviation (*n* = 3); Different superscript letters in a row means significant differences among values (*p* < .05).

Abbreviation: n.d.: not detected.

### 3.7 Vitamin C

Vitamin C (ascorbic plus dehydroascorbic acid) ranged between 819 (L3) and 1143 mg kg^−1^ fw (WT) (Table 3), whereas most samples showed amounts between 956 and 1018 mg kg^−1^ fw with no significant differences among them.

### 3.8 Antioxidant activity

Values regarding the antioxidant activity of IPL are reported in Table 3. The antioxidant activity measured through the ABTS assay ranged between 3686 and 8838 mg TE kg^−1^ dw. The highest values were found in wild plants (8641 and 781.6 mg TE kg^−1^ dw) and cultivated C2 and C3 (8584 and 8047 mg TE kg^−1^ dw), and L3 and L4 (8838 and 8299 mg TE kg^−1^ dw), lacking significant differences among them. Cultivated C1 and L2 plants (7163 and 6902 mg TE kg^−1^ dw) showed intermediate values, and the lowest antioxidant activity measured by the ABTS method was found in L1 plants (3686 mg TE kg^−1^ dw).

DPPH values showed a similar trend; the highest ones were found in WU, C2, and C3 samples (4020, 3614, and 3758 mg TE kg^−1^ dw) or L3 and L4 (3894 and 3669 mg TE kg^−1^ dw). WT, C1, and L2 samples (3084, 3336, and 3405 mg TE kg^−1^ dw) showed intermediate values, and the significantly lowest antioxidant activity measured by the DPPH method was found in L1 plants (1233 mg TE kg^−1^ dw).

### 3.9 Antiproliferative and toxic activities of IPL extracts against HT‐29 and CCD‐18 cells

**FIGURE 3:**
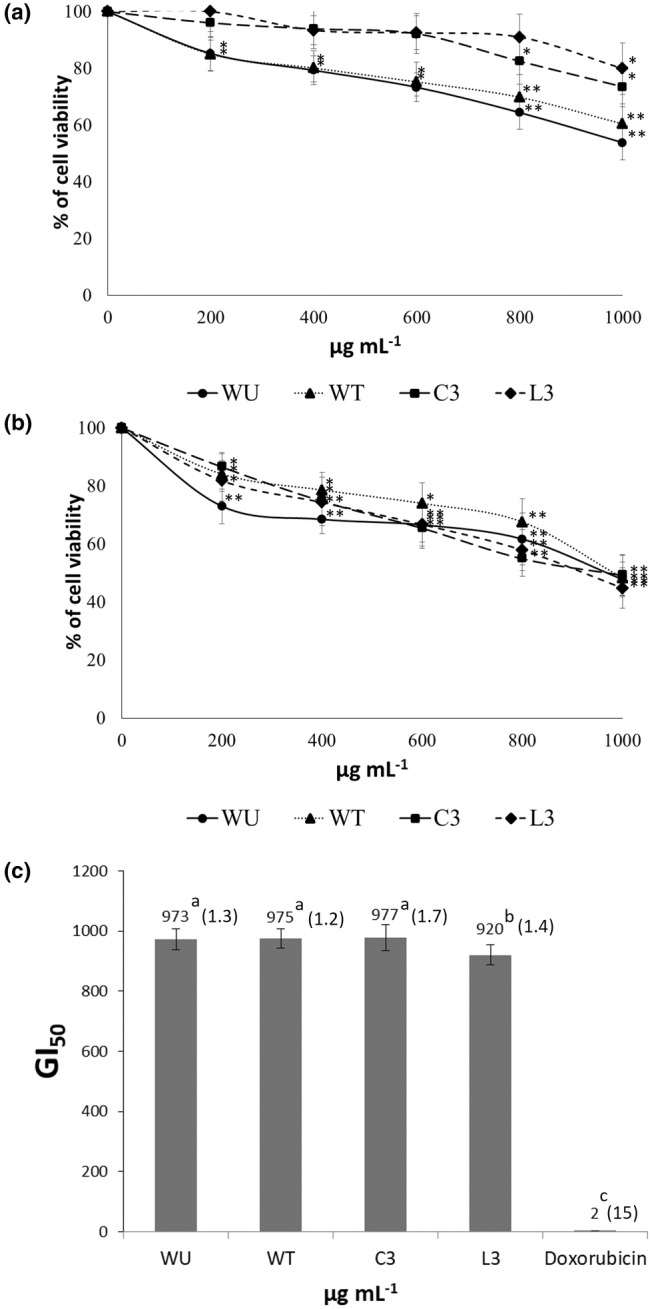
Dose–response curves of HT‐29 cells viability after treatment with different concentrations of hydroalcoholic leaf extracts from wild (WU and WT) and cultivated (C3 and L3) *M. crystallinum*. Extracts were assessed after 48 h (a) and 72 h (b) of cell exposure to plant extracts with statistical significance equal to *p < .05; **p < .001. (c) GI_50_ values displayed by WU, WT, C3, and L3 plant extracts compared with that of doxorubicin. Data represent the mean of three complete independent experiments ± SD (error bars). In a bar, means followed by different lower‐case letters are significantly different at *p* < .05. The Selectivity Index (SI) for HT‐29 vs. CDD‐18 cells exposed to IPL extracts (see Material and Methods section) is shown in parentheses in (c).

The hydroalcoholic extracts from IPL having the highest antioxidant activity (WU, WT, C3, and L3) were assessed for their antiproliferative activity against HT‐29 cells, and cell growth inhibition was observed in all cases (Figure 3a,b). After 72 h of cell exposure to IPL extracts, GI_50_ (50% of cell growth inhibition) was reached for all extracts, ranging from 920 to 977 μg mL^−1^ (Figure 3c). The SI of HT‐29 versus normal CCD‐18 cells was assessed after 72 h of cell exposure to IPL extracts, and such an index ranged from 1.2 (WT) to 1.7 (C3).

## 4 DISCUSSION

### 4.1 Effects of fertigation and light treatment on growth parameters and moisture content in cultivated *M. crystallinum*

#### 4.1.1 Fertigation parameters

The EC values of drainage in experiment 1 were higher than those acceptable for other crops (Moya et al., 2017; Urrestarazu et al., 2008), confirming the halophytic character of *M. crystallinum* (Atzori et al., 2017). In experiment 1, a higher metabolic activity in the roots due to nutrient absorption proportional to the uptake of water was noticed, while the EC of the drainage of treatments C2 and C3 was higher due to a greater concentration of salts in the fertigation solution (Chourak et al., 2022). In experiment 2, the absorption of fertigation solution and the ratio of water to salts, expressed through the EC, showed a significantly better performance of the horticultural LED lamps (L2, L3, and L4) than the control lamp (L1) (Gallegos‐Cedillo et al., 2018).

#### 4.1.2 Fertigation uptake and growth parameters

Plants accumulate organic and inorganic solutes in the cytosol to raise the osmotic pressure and to maintain turgor and the driving gradient for the uptake of water and mineral nutrients (Rhodes & Samaras, 1994). The spectral composition of light not only alters biomass growth but also the functionality of stomata and the overall water use of plants (Pennisi et al., 2019).

Nitrate and potassium are used as indicators of adequate mineral uptake in a crop (Ferrón‐Carrillo et al., 2021). In experiment 1, water uptake fit a slightly modified model of Sonneveld and Voogt (2009) for salinity‐tolerant plants. The effect of nitrate and potassium uptake from the nutrient solution on their transport in the xylem of plants was previously described with tomato plants (Gallegos‐Cedillo et al., 2016), where these values were optimal and remained constant in the nutrient solution between 3.5 and 4.5 dS m^−1^, while for ice plants, they remained constant from 4 to 6 dS m^−1^.

None of the saline treatments negatively affected ice plant mass growth compared to the control. In contrast, the better fresh mass yields at higher saline levels may suggest a kind of growth stimulation induced by salinity. Our results agree with those of other authors, as they found no significant differences in *Mesembryanthemum* plant biomass grown at elevated EC (Atzori et al., 2017). The ice plant is a strict halophyte species that maintains its optimum growth up to values of at least 6 dS m^−1^ or even higher saline levels (Figure S4) (Atzori et al., 2017), which highlights its potential for saline agriculture.

Light spectrum and intensity are important for crop growth, as each spectral range influences a specific plant receptor (Spalholz et al., 2020). Plants showed the highest absorptions when using the NS1 lamp, with a broad‐spectrum equivalent to sunlight and a longer wavelength in blue, responsible for vegetative growth, and AP67, responsible for vegetative growth and flowering, and a longer wavelength in red and far red. Similar results were found by Haddaji et al. (2023), where basil performed best in blue light and *Trigonella foenum‐graecum*, *Anethum graveolens*, and *Anthriscus cerefolium* plants in red light. Ohashi‐Kaneko et al. (2007) reported lower nitrate levels in Komatsuna plants grown under red fluorescent light than plants grown under sunlight, although no significant differences in nutrient uptake were found under the same light treatments, indicating that plant response to light may also depend on the crops and cultivars involved.

In experiment 2, the values of ice plant growth agreed to results published by Haddaji et al. (2023) for three basil cultivars using the same continuous light‐emitting diodes compared to control white LED lamps. Nájera and Urrestarazu (2019) studied the effects of LED light intensity and spectral quality on six vegetables and concluded that the use of the AP67 lamp contributed to a significant increase in fresh mass compared to the control (T1, white Roblan).

### 4.2 Fatty acids

FA profiles of IPL have been scarcely reported with contradictory results. For instance, IPL collected in the South of Portugal showed high proportions of SFA (26.1% PA, 12.0% 22:0, 10.5% 20:0), as well as 13.8% OA and 19.6% LA, whereas ALA was not detected (Vizetto‐Duarte et al., 2019). In contrast, cultivated IPL contained mainly ALA (48.0%–54.8% of total FA), PA (18.1%–24.9%), and LA (12.3%–13.2%) (Nouairi et al., 2006; Oliveira‐Alves et al., 2023). This way, the influence of plant type (wild or cultivated) and growth conditions on the FA profiles of IPL remains unclear. Marked differences in the FA profiles between wild and cultivated specimens were found in the current study. The ALA proportion was significantly higher in wild plants, probably in response to environmental temperatures. Wild plants are exposed to lower temperatures than cultivated ones, which were grown at a constant temperature of 20°C–22°C. It is known that the fluidity of cell membranes can be maintained at lower temperatures by increasing the levels of unsaturated FA in the chloroplast lipids through the regulated activity of FA desaturases, which allows the plants to maintain their photosynthetic functions (Upchurch, 2008).

Saline levels did not significantly affect ALA percentages among cultivated plants. *M. crystallinum* tolerates high salinity, and irrigation solutions of up to 35 dS m^−1^ had no adverse effects on biomass production and succulence (Atzori et al., 2017). The EC levels assayed in the current work were far below such a limit and therefore could not have been enough for the plant to be considered an abiotic stress factor.

Light treatment significantly affected the FA profiles of leaves among cultivated plants. L1 specimens (control treatment) showed significantly lower and higher proportions of SFA (mainly PA) and PUFA (mainly ALA) than the other lighting treatments. In contrast, higher SFA and lower PUFA (particularly ALA) proportions were found in L3 and L4 plants. Exposure to shorter wavelength radiation, particularly UV, is considered as a potential abiotic stress factor (Shi & Liu, 2021). Plants can activate the octadecanoid signaling pathway in response to stress conditions, where ALA is the initial substrate of a cascade of enzymatic reactions leading to the synthesis of oxylipins such as jasmonic acid and related metabolites, which are associated with signal transmission against plant stress (Singh et al., 2022). This way, high proportions of ALA would be expected in highly exposed leaves to UV radiation, as observed in the current work, which would be available as substrate for the octadecanoid pathway.

ALA is an essential *n*‐3 PUFA that must be provided with the diet (Yuan et al., 2022). This way, IPL, particularly those from wild specimens, can be considered dietary ALA suppliers.

### 4.3 Total carotenoids

Carotenoids are organic pigments acting as free radical scavengers in the plant metabolism with bioactivity against several disorders induced by oxidative stress (Monego et al., 2017). Some carotenoids (α‐carotene, β‐carotene, and β‐cryptoxanthin) have provitamin A activity, and their supply is required to maintain visual function.

It has been reported that there is a decrease in carotenoid levels in many plants in response to saline stress, even in some halophytes (Uarrota et al., 2018). However, the current work showed a positive correlation in cultivated plants between total carotenoids and salinity, being more significant at the highest assayed level (6.0 dS m^−1^). This trend has also been reported in previous studies on *M. crystallinum* (Atzori et al., 2017; He et al., 2022).

The high concentration of carotenoids found in IPL in response to increased salinity is probably related to the activation of the carotenoid synthetic pathway. In this regard, the production of reactive oxygen species (ROS) is increased in plants exposed to high salinity, and the biosynthesis of carotenoids may be promoted as a protective mechanism (Uarrota et al., 2018). The expression of genes codifying the enzymes involved in the carotenoid biosynthesis pathway (such as S*n*PSY2 and S*nβ*‐LCY, codifying for phytoene synthase and lycopene β‐cyclase, respectively) was enhanced in *Solanum nigrum* and *Solanum lycopersicon* under salt stress (Ben Abdallah et al., 2016). This mechanism could also work in *M. crystallinum*.

The effects of light quality on carotenoid concentration in IPL have not been extensively assessed, and the available data in the literature are not conclusive (Badmus et al., 2022; Dhami & Cazzonelli, 2020). Carotenoid levels in IPL exposed to different light sources in this study ranged within a narrow interval (65–88 mg kg^−1^ fw), which agrees with previous works; plants grown indoors under several combinations of red and blue LED lights showed carotenoids concentrations ranging 30–72 mg kg^−1^ fw (He et al., 2017, 2022; Zhang et al., 2021).

### 4.4 Phenolic compounds

Qualitative and quantitative profiles of phenolic compounds in plants are highly dependent on many biotic and abiotic variables, and the influence of the extraction and analysis methods should be considered. This way, it is complex to compare the results of different studies regarding the occurrence of phenolic compounds in plants.

Wild plants show typically higher TPC than cultivated specimens, which can be explained considering that phenols are synthesized by plants in response to stress conditions, and wild plants are more exposed to such conditions than their cultivated counterparts, which receive a controlled supply of light, water, and nutrients. Among cultivated plants, the highest TPC was found in L4 specimens. Although it has been described that blue and UV light usually promote phenol synthesis in plants through the regulation of gene expression in response to a larger production of ROS induced by such radiation, this fact has not been extensively confirmed. For instance, Zhang et al. (2021) found no significant differences in TPC in cultivated IPL under different light treatments. Indeed, these authors reported higher TPC in leaves subjected to 85% red light and 15% blue light than in leaves under 100% blue light. Other studies highlighted the influence of the cultivar, as different cultivars of *Chrysanthemum morifolium* showed different TPC in response to light treatment (white, red, blue, or red:blue 75:25) (Zheng & Van Labeke, 2017). Overall, it appears that the type of lighting to which plants are exposed is not a defining variable per se regarding TPC, and many other biotic and abiotic factors are more related to this outcome.

No significant difference in TPC was observed within cultivated plants under different saline conditions in this study, which may be due to the high saline tolerance of *M. crystallinum*. Atzori et al. (2017) found no significant differences in TPC in IPL irrigated with highly saline solutions (up to 35.0 dS m^−1^), while Falleh et al. (2012) reported no variation in the amount of TPC in shoots of *M. edule* grown with saline solutions of 300 and 600 mM NaCl (equivalent to around 27 and 55 dS m^−1^ respectively), although a TPC decrease was noticed compared with shoots from plants cultivated with a no‐saline solution. Other authors reported an increase in TPC from 35 to 275 mM NaCl (~3 to 25 dS m^−1^) and a further decrease from 275 to 465 mM NaCl in *M. nodiflorum* grown between 35 and 465 mM NaCl (equivalent to around 3 and 42 dS m^−1^) (Lima et al., 2021).

The estimated TPC in IPL was 3–4‐fold higher than that of TFC in most analyzed wild and cultivated specimens. This finding agrees with those from leaves of wild *M. crystallinum* extracted with boiling water (TPC/TFC = 4.9) (Ibtissem et al., 2012), and also with those from cultivated *M. crystallinum* extracted with ethanol:water 80:20 v/v (TPC/TFC = 4.2) (Oliveira‐Alves et al., 2023).

The phenolic profiles of IPL (Table 4) have been scarcely explored. The parameters for the identification of compounds are given in Table S2. Previously, phloretin, quercitrin, and avicularin were identified as the main phenolic compounds in methanol extracts of IPL (Hanen et al., 2009). In contrast, other phenolic compounds were found in ethanol‐water extracts, such as *p*‐coumaric acid, apigenin, 2‐*O*‐(*p*‐coumaroyl)‐1‐malic acid, diosmin, luteolin, *p*‐coumaric, and sinapic acid derivatives, and chrysin‐6‐C‐glucosyl‐8‐C‐arabinoside (Calvo et al., 2022; Oliveira‐Alves et al., 2023). According to these data, the methanol extracts of *M. crystallinum* would contain mainly flavonoids, whereas the presence of phenolic acids is more notorious in hydroalcoholic extracts. Such evidence agrees with the results found in the current study, as phenolic acids were the main compounds identified in all analyzed extracts from both wild and cultivated samples, with eight compounds. In addition, two flavonoids, gallocatechin (−) and epicatechin gallate (−) were detected, although the last occurs in very low amounts and only in wild plants.

### 4.5 Vitamin C

Vitamin C has powerful antioxidant activity and is a cofactor for several enzymes in the human metabolism, besides its role in several human physiological processes such as iron absorption or collagen synthesis (Paciolla et al., 2019). Vitamin C is supplied with the diet as the human organism is unable to synthesize it, being 75–90 mg the recommended daily intake (Paciolla et al., 2019). According to the results of this work, a portion of 100 g of fresh IPL can fulfill the recommended daily allowance of vitamin C, regardless of the plant type (wild or cultivated).

Although some differences can be noted regarding the vitamin C content of IPL in this work, particularly in C2 and L3, there is not a clear relationship between treatments. A previous study did not find significant differences in the vitamin C content between wild and cultivated *M. nodiflorum*, which was ~450 mg vitamin C kg^−1^ fw (Castañeda‐Loaiza et al., 2020).

Cultivated *M. crystallinum* at different salinity exposures showed a significant increase in vitamin C when the conductivity increased from 1.8 to 25 dS m^−1^, but not when it increased from 1.8 to 13.7 dS m^−1^ (Rodríguez‐Hernández & Garmendia, 2022). Thus, vitamin C amounts can increase with high salinity exposure, and the salinity range assayed in the current work was probably not large enough to induce saline stress in ice plants.

### 4.6 Antioxidant activity

Phenols prevent ROS‐induced stress in plants, and a positive relationship is usually found between the phenolic content and the antioxidant activity of plant extracts (Calvo et al., 2022). The ABTS and the DPPH assays were used in this work to determine the antioxidant activity of extracts through their radical scavenging ability, given their high sensitivity (Lima et al., 2021).

The ratio between the antioxidant activity measured by the ABTS and DPPH ranged in most samples between 2.0 and 2.5, which may be explained considering that the ABTS^•+^ radical is more soluble in organic solvents and in water than DPPH^•^; thus, the ABTS assay could be more sensitive when measuring the antioxidant activity of water‐soluble compounds such as phenols (Pathiraja et al., 2023).

Antioxidant activity of 84 and 21 mmol TE kg^−1^ dw (21,020 and 5269 mg TE kg^−1^ dw using the ABTS and DPPH assays, respectively) was reported in hydroalcoholic extracts (50% ethanol) of cultivated IPL (Calvo et al., 2022). Lower DPPH values were found using 80% methanol extracts of cultivated *M. crystallinum* under different combinations of red and blue light treatments: between 25 and 33 mg TE kg^−1^ fw, resulting in 830–1100 mg TE kg^−1^ dw considering the moisture of leaves found in that study (Kim et al., 2021). Such values are higher and lower than those found in the current study using the DPPH assay with 60% methanol extracts. It is important to note that the type of extraction solvent may influence the antioxidant capacity of the extracts (Cybulska et al., 2021; Laosirisathian et al., 2020). This fact, together with the influence of biotic and abiotic factors during plant growth, can explain the differences in the antioxidant activity of IPL extracts among studies.

The antioxidant activity of several species from the *Mesembryanthemum* genus, such as *M. edule* and *M. nodiflorum*, has been reported considering the concentration of the plant extract able to cause a 50% inhibition (IC_50_, which is the concentration of an antioxidant‐containing solution required to scavenge 50% of the initial DPPH or ABTS radicals), and such results are therefore not comparable with those of the current study (Castañeda‐Loaiza et al., 2020; Hanen et al., 2009; Lima et al., 2021).

### 4.7 Correlation among parameters

**FIGURE 4:**
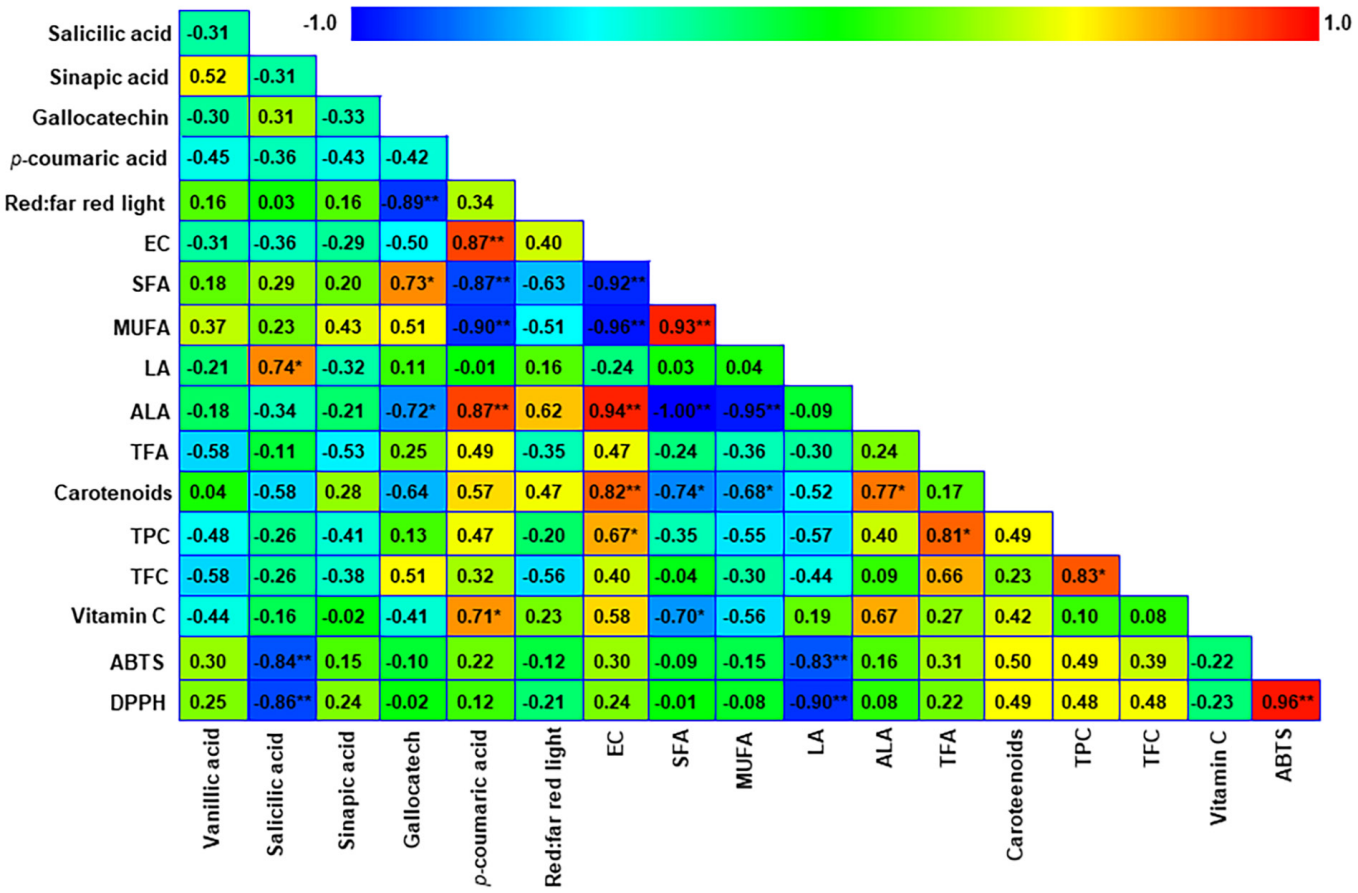
Heat map of the correlations between the different variables. The redder colors indicate a stronger and positive correlation, while the bluer colors indicate a stronger and negative correlation. Correlations were evaluated with a significance of *p* < .05 (*) and *p* < .001 (**).

Figure 4 shows the correlations between the antioxidant activity, growth parameters, and bioactive compounds in IPL samples. Both antioxidant methodologies have a high and positive correlation. TFC, TPC, and carotenoids showed a positive correlation with both DPPH and ABTS, indicating their contribution to the measured antioxidant activity, although values lack statistical significance. As previously discussed, EC and carotenoids have a positive correlation. A positive and significant correlation between ALA and EC was observed, while the correlation of EC with both SFA and MUFA was negative. This fact has been previously reported in another halophyte, *Suaeda salsa*, and is interpreted considering that an increased concentration of unsaturated FA in membrane lipids enhances the tolerance of photosystem II to salt stress (Sui et al., 2010). Another positive and strong correlation was found between EC and *p*‐coumaric acid. This phenolic acid was found in high salinity‐tolerant rice, and its content was strongly increased under salinity stress given its high radical scavenging activity (Minh et al., 2016). Moreover, the correlation between EC and TPC was significantly positive. Other correlations were as expected, for instance, the negative correlation between SFA and ALA. Altogether, the commented correlations suggest that IPL constitutes a nutrient system of high phytochemical value, easily and cheaply obtained in marginal soils of high EC, which could constitute a valuable resource for farmers while providing its potential consumers with a vegetable of high functional value.

### 4.8 Antiproliferative activity of IPL extracts against HT‐29 and CCD‐18 cells

The anticancer potential of some halophytes has been recently published (Custodio et al., 2022), but studies on the antiproliferative potential of IPL extracts on cancer cells are scarce. Methanol extracts of IPL were efficient in decreasing cell growth in MCF7‐1 (human breast carcinoma) and H1299 (human lung carcinoma) (GI_50_ of 38.3 and 22.1 μg mL^−1^, respectively) (Essa & Elsebaie, 2018). The main phenols identified in such extracts were catechin and pyrogallol (41.1% and 7.3% of total phenols). Conversely, other authors found that an ethanol extract of IPL assayed at 500 μg mL^−1^ reached 80% cell viability after 48 h exposure in the HCT116 human colon cancer cell line, measured by the MTT assay, and therefore the GI_50_ was not achieved (Seo & Ju, 2019). In both cited studies, a dose‐dependent response was noticed.

To our knowledge, the antiproliferative activity of IPL extracts on HT‐29 cells (human colorectal adenocarcinoma) has not been reported yet. The MTT assay showed concentration‐ and time‐dependent inhibitory effects on HT‐29 cells for all assayed extracts after 48 and 72 h of exposure (Figure 3a,b). Cell viability at 72 h at the highest concentration tested (1000 μg mL^−1^) for the different IPL samples was 5%–35% lower than that obtained at 48 h. After 72 h culture, cell growth inhibition was exercised in a similar way by all samples, which reached GI_50_ between 920 (L3) and 977 (C3) μg mL^−1^ of extract (Figure 3c). It is necessary to consider that GI_50_ values were not associated with antioxidant activity values or a specific phenolic profile, and all extracts exerted similar bioactivity against HT‐29 cells. Therefore, the inhibition of cell growth noted would be due to a variety of causes, such as the occurrence of phenolics along with other undetermined compounds in the extracts, and all of them would probably act synergistically to achieve the antiproliferative effects measured.

These results suggest that HT‐29 cells are responsive but not very sensitive to hydro‐methanol extracts of IPL. Additionally, the SI of HT‐29 versus normal CCD‐18 cells was assessed. Extracts with SI > 2 are highly selective against cancer cells, while those with SI < 2 exercise toxicity to normal cells (Vichitsakul et al., 2023). SI for herbal drugs or pure compounds is critical to deciding possible deeper research on the subject (Peña‐Morán et al., 2016). The SI at 72 h ranged from 1.2 (WT) to 1.7 (C3), from which it can be concluded that IPL extracts were moderately selective against HT‐29 cells. Further research focused on the purification of the phenolic fraction of IPL and the assessment of its potential antitumor properties could shed light on this issue, considering that extracts contain not only phenols but also probably polysaccharides and other polar compounds that could influence the overall bioactivity against HT‐29 cells.

## 5 CONCLUSIONS

This work shows the influence of growth parameters on the phytochemical composition of IPL. Wild specimens showed significantly lower proportions of SFA and MUFA and a higher proportion of ALA than their cultivated counterparts, which is nutritionally advantageous. Among cultivated plants, increased EC led to a higher amount of total carotenoids, ALA, *p*‐coumaric acid, and TPC, thus improving the concentration of valuable phytochemicals. The positive correlation of EC with *p*‐coumaric acid was due to the effect that salinity stress induces in halophytes, given the high radical scavenging activity of this phenolic compound. Moreover, exposure to NS1 and AP67 lamps was found to be more efficient than other lamps to increase TPC, carotenoids, and antioxidant activity. Interestingly, NS1 and AP67 lamps also led to significantly higher values of fresh plant mass than those obtained in the control experiment (L18 T8 lamps). Therefore, the results with cultured plants suggest that plants having similar functional value to wild ones can be obtained by fine‐tuning some easily manipulated parameters, such as EC and the type of lamp used in the culture chamber. All assayed IPL extracts exercised time‐ and dose‐dependent antiproliferative activity against HT‐29 cells, reaching GI_50_ values slightly lower than 1000 μg mL^−1^. Due to the marked influence of the extraction solvent on the phytochemical composition of the resulting extract, further research to test the effect of different extraction solvents on both phenolic profiles and antitumor activity would be desirable. Furthermore, in vitro experiments on the antitumor activity of IPL against other cancer cell lines and in vivo experiments for checking the health status of IPL‐fed animals could constitute other interesting lines of research. Finally, it is expected that through the application of the discoveries described in this work, some minority sectors of the agricultural system will be promoted, such as those that use saline soils of little value for the development of conventional crops.

## AUTHOR CONTRIBUTIONS

**Miguel Ángel Rincón‐Cervera:** Conceptualization (equal); data curation (equal); formal analysis (equal); investigation (equal); software (equal); validation (equal); visualization (equal); writing – original draft (equal). **Tatiana Pagan Loeiro da Cunha‐Chiamolera:** Data curation (equal); formal analysis (equal); investigation (equal); methodology (equal); software (equal); visualization (equal); writing – original draft (equal). **Tarik Chileh‐Chelh:** Data curation (equal); formal analysis (equal); investigation (equal); methodology (equal); software (equal); validation (equal); visualization (equal). **Minerva Carmona‐Fernández:** Data curation (supporting); formal analysis (supporting); validation (equal); visualization (supporting). **Miguel Urrestarazu:** Investigation (equal); methodology (equal); resources (equal); visualization (equal). **José Luis Guil‐Guerrero:** Conceptualization (lead); data curation (lead); formal analysis (equal); funding acquisition (lead); investigation (lead); methodology (lead); project administration (lead); resources (lead); software (equal); supervision (lead); validation (equal); visualization (equal); writing – review and editing (lead).

## FUNDING INFORMATION

Project P_LANZ_2023/003 (Vicerrectorado de Política Científica, University of Almeria), Project P20_00806 (Junta de Andalucía), and the program “Ayudas para Captación, Incorporación y Movilidad de Capital Humano de I+D+i (PAIDI 2020)” (Andalusia Ministry of Economic Transformation, Industry, Knowledge, and Universities).

## CONFLICT OF INTEREST STATEMENT

The authors declare that they do not have any conflict of interest.

## ETHICAL REVIEW

This study does not involve any human or animal testing.

## Supporting information


Figure S1

Figure S2

Figure S3

Figure S4

File S1

Table S1

Table S2

Data S1

## Data Availability

All data used to prepare this article is included in the tables and figures.
