# Supplementary figures and images for: Growth parameters, phytochemicals, and antitumor activity of wild and cultivated ice plants (Mesembryanthemum crystallinum L.)
Source: Food Sci Nutr. 2024 Jun 21;12(9):6548–62. doi: 10.1002/fsn3.4286 (PMC11561852; doi:10.1002/fsn3.4286)

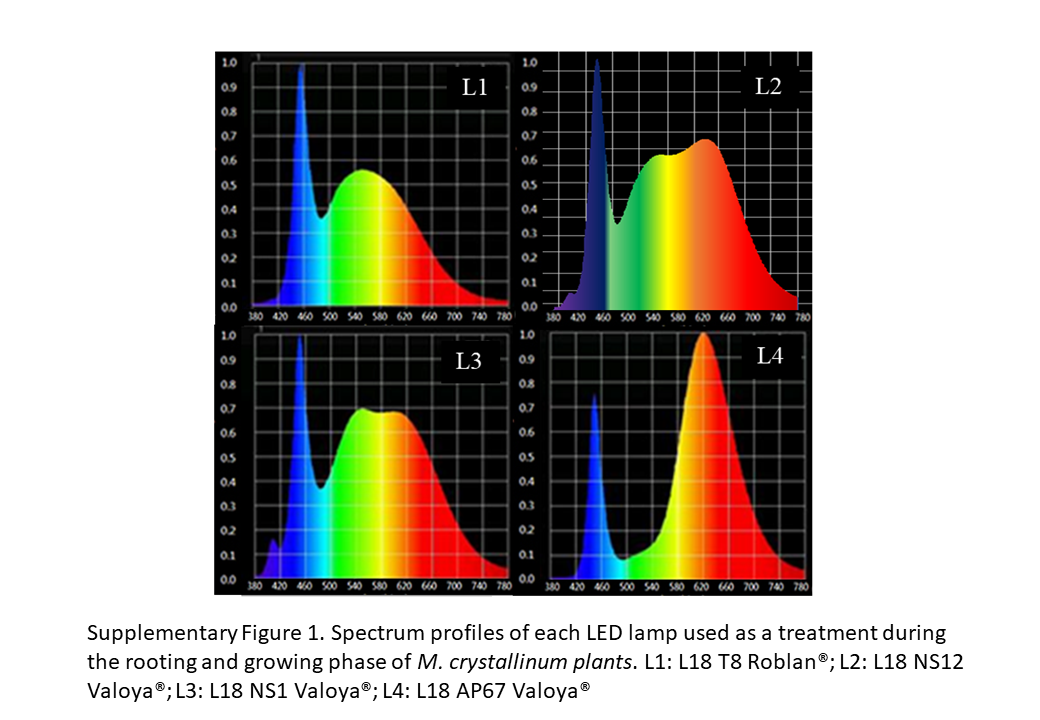

Supplement: Supplementary file 1 — Figure S1 [file FSN3-12-6548-s001.tif]

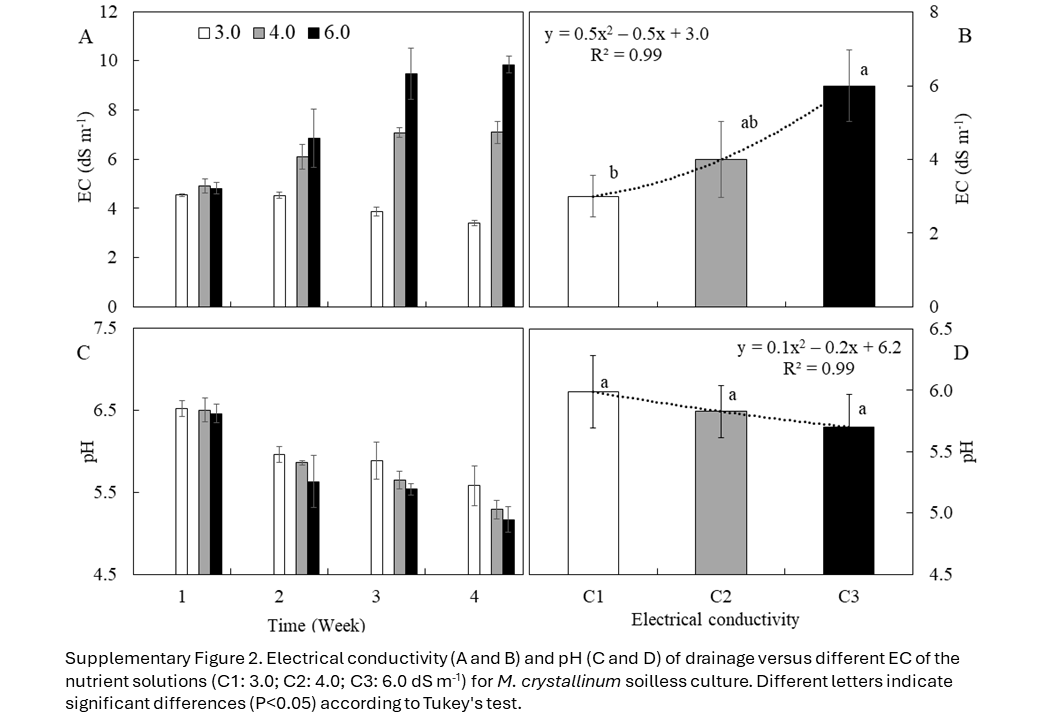

Supplement: Supplementary file 2 — Figure S2 [file FSN3-12-6548-s008.tif]

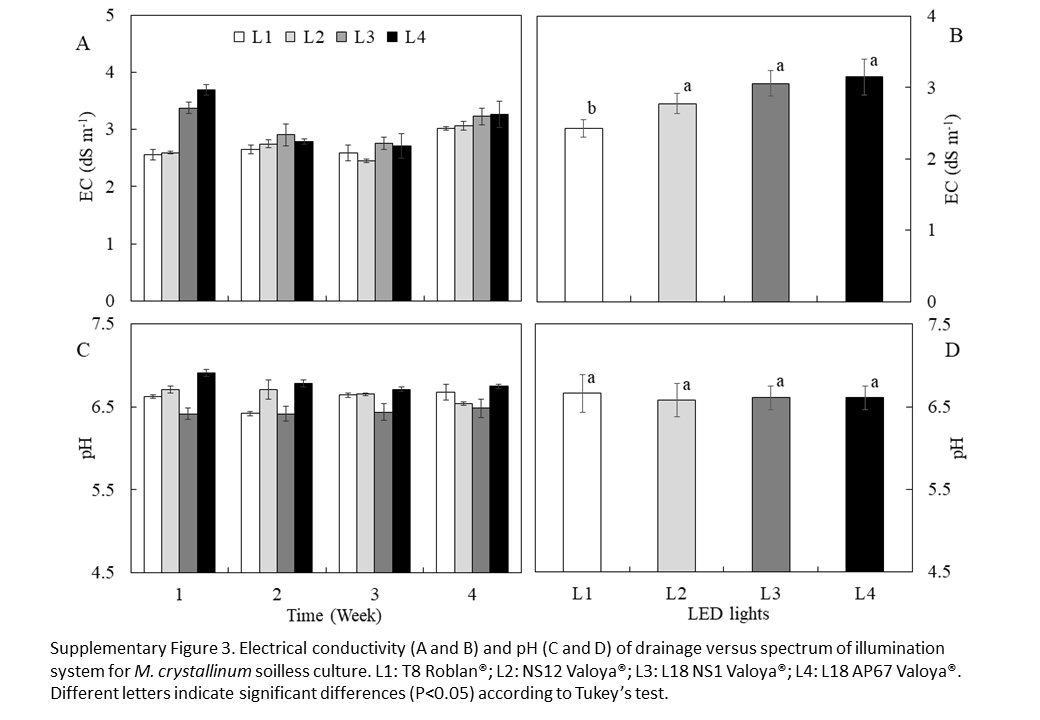

Supplement: Supplementary file 3 — Figure S3 [file FSN3-12-6548-s006.tif]

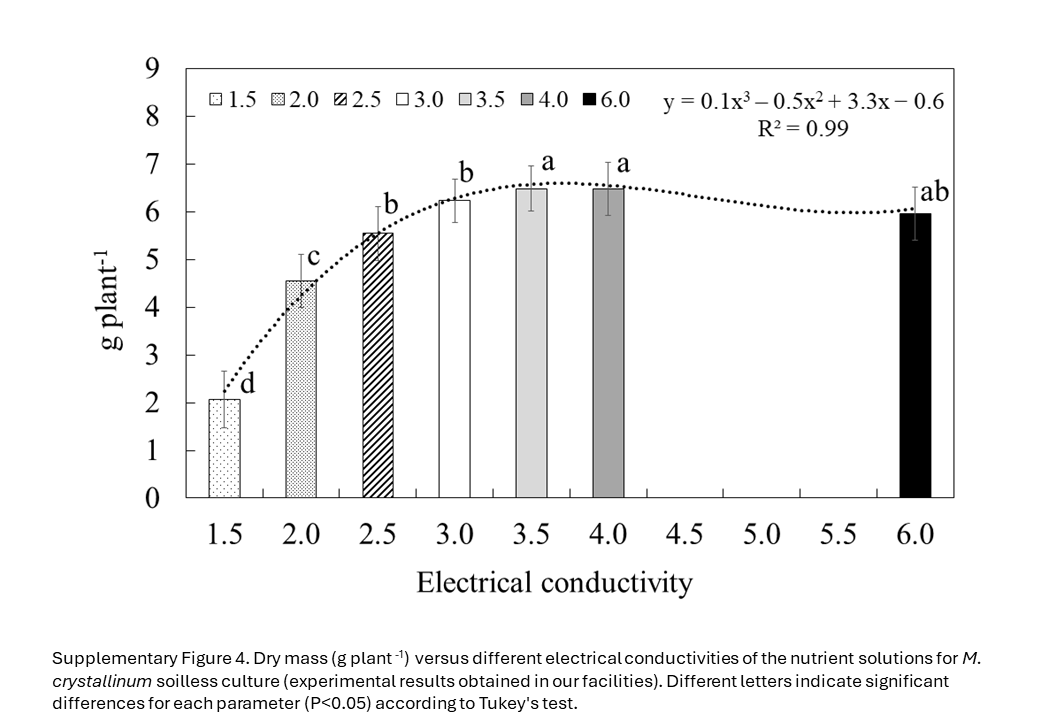

Supplement: Supplementary file 4 — Figure S4 [file FSN3-12-6548-s005.tif]
