# Supplementary material for: Growth parameters, phytochemicals, and antitumor activity of wild and cultivated ice plants (Mesembryanthemum crystallinum L.)
Source: Food Sci Nutr. 2024 Jun 21;12(9):6548–62. doi: 10.1002/fsn3.4286 (PMC11561852; doi:10.1002/fsn3.4286)
Supplement: Supplementary file 5 — File S1 [file FSN3-12-6548-s007.pdf]

Supplementary File 1. Description of Methodologies

1. Growth, fertigation, and lighting conditions applied to cultivated *Mesembryanthemum crystallinum*

M. crystallinum plants were grown under two independent experiments. The first one used three treatments with different electrical conductivities in the nutrient solution, 3.0 (C1), 4.0 (C2) and 6.0 (C3) dS m^{-1}. A Roblan® L18 T8 white LED lamp (Toledo, Spain) was used for the test. In the second experiment, four LED lamps were evaluated: L18 T8 Roblan® as control (L1), and other lamps that are specifically manufactured for lighting technology purposes in horticulture: L18 NS12, L18 NS1 and L18 AP67 Valoya® (Helsinki, Finland) as treatment 2, 3, and 4, respectively (L2, L3, L4).

For both experiments, three light-emitting diode (LED) lamps were used, both on a surface of 0.504 m^2. All lamps had the same length and power (18 W). The spectra of each treatment were measured with an UPRtek MK350S LED (UPRtek, Taiwan). LP471-PHOT and LP471-PAR sensors (Delta OHM®, Padua, Italy) were used to measure luminance (lux) and photosynthetic photon flux, PPF (mmol m^{-2} s^{-1}), as described by Ferrón-Carrillo *et al*. (2021).

The application of the fertigation was performed when the water in the culture unit reached 10% of the readily available water, and the necessary volume was added to obtain between 15-25% drainage (Rodriguez *et al*., 2014; Peçanha *et al*., 2021). For each treatment, two controls were established as fertigation controls, consisting of a control dripper and a drainage tray that served as measurement and monitoring points for the fertigation supplied and the uptake response. The volume, pH, electrical conductivity (EC), nitrate and potassium content of the supplied nutrient solution and drainage were measured daily in each pot with a container that was adapted to its morphology. The pH and EC were monitored using systems from HORIBA Ltd. (LAQUAact PC110-K). The contents of nitrate and potassium were measured with systems from HORIBA Ltd. Japan (LAQUAtwin B-741 and LAQUAtwin B-731, respectively). The absorption of nitrates and potassium in mmol·$plant^{-1}$ was quantified based on the balance between input and output (Urrestarazu *et al*., 2008). Volume (L) was measured using a test tube graduated to the hundredth of a millimeter.

To perform the correlations between the determinant parameters with the rest of the phytochemical parameters in each experiment, the EC was considered in the salinity essay and in the illumination essay ratio red:far red. The EC of the substrate and of the soil where the plants were cultivated were measured using saturated extract techniques (Peçanha *et al.*, 2021), while the red:far red ratio was measured as described by Najera and Urrestarazu (2019).

2. Evaluation of growth parameters

The evaluation of growth parameters was carried out 30 days after transplantation. The experimental unit was four plants per treatment and four replicates of each treatment. The plants were divided by their different organs; the fresh weight of roots, stems and leaves was obtained, then the dry weight was obtained by placing the material in an oven (Thermo Scientific Heratherm, Germany) at 85 °C until achieving a constant weight. A precision analytical balance (Adventurer Analytical OHAUS Modelo AX 124/E, USA) was used, expressing the result as g plant^{-1}.

Plants were collected at baby leaf stage (16-17 leaves). After harvesting, plants were stored in thermal bags and frozen at -24 ºC until processing. Once in the laboratory, the greens were labeled, weighed, measured, and placed in a glass desiccator until analysis.

3. Fatty acids

Fatty acid profiles were obtained after direct derivatization to fatty acid methyl esters (FAME). Dry leaves (~500 mg) were finely minced and placed in 10-mL glass tubes. Then, 1 mL of n-hexane and 2 mL of a methylating mixture (methanol:acetyl chloride 20:1 v/v) were added. Tubes were capped and heated at 100 ºC for 30 min in a hot block, then cooled at room temperature. After adding 1 mL of distilled water, tubes were centrifuged at 2,000 g for 5 min and the upper hexane layer was collected for analysis. FAME were analyzed in a Focus GC (ThermoElectron, Cambridge, UK), equipped with a flame ionization detector (FID) and an Omegawax 250 capillary column (30 m × 0.25 mm i.d. × 0.25 μm film thickness; Supelco. Bellefonte, USA), as previously described (Lyashenko *et al.*, 2019). Peaks were identified according to their retention times compared to a FAME standard mix (Supelco 37 Component FAME Mix, Sigma-Aldrich).

4. Total phenols and total flavonoids

Extraction of phenolic compounds was carried out according to Lyashenko *et al.* (2021) with some modifications. Fresh leaves (1 g) were finely minced and extracted three times with 3 mL of methanol:water 60:40 v/v. After centrifuging (1,000 g, 10 min) the supernatants were collected, combined, and evaporated at 60 °C with nitrogen until a final volume of 1 mL. The extract was filtered (0.45-mm membrane filter, Millipore, Durapore®, Ireland) before analysis. Total phenolic content (TPC) was measured using the Folin-Ciocalteu (F-C) assay developed by Singleton *et al.*, (1999) with minor modifications. Briefly, 10 µL of phenolic extracts 0.79 mL of MiliQ water and 50 µL of Folin-Ciocalteu reagent were mixed, vortex and allowed to stand for 5 min at room temperature. Next, 150 µL of a 20% sodium carbonate solution were added and vortex. A control sample was also prepared. After incubation at room temperature for 2 h in darkness, the absorbance of the mixture was read at 765 nm on a UV-VIS spectrophotometer. Deionized water was used as blank. Results were expressed as mg of gallic acid equivalents (GAE) per 100 g of sample using a standard curve of gallic acid (0-500 mg/100 mL). Determinations were carried out in triplicate.

Total flavonoid content (TFC) of the phenolic extract was determined using the aluminum chloride method according to Zou *et al.*, (2004) with some modifications. Briefly, 0.5 mL of the phenolic extract was collected and 150 µL of 5% $NaNO_2$ solution was added. After 5min, 150 µL of 10% $AlCl_3$ solution was added to the mixture, which was kept at room temperature for 5 minutes, followed by the addition of 0.7 mL of 1M NaOH. The absorbance was then immediately measured at 510 nm on a UV-VIS spectrophotometer. Deionized water was used as blank. The results were expressed as mg of quercetin equivalents (QE) per 100 g of sample using a standard curve of quercetin (0-200 mg/100 mL). Determinations were carried out in triplicate.

5. Identification of phenolic compounds

The chromatographic separation was performed on a Vanquish Flex Quaternary LC equipped with a reverse-phase C18 column (Hypersil Gold, 100 mm × 2.1 mm, 1.9 µm) (Thermo Fisher Scientific, San Jose, CA, USA) at flow rate of 0.2 mL/min. The compounds were separated with gradient elution using acidified water (H_2O containing 0.1% formic acid and 4 mM ammonium formate) (A) and methanol (B) as eluents at room temperature (25 ºC). The step gradient was as follows: 0-1 min 95% of A; then, it was linearly decreased to 70% in 7 min, to 50% in 5 min and remained constant during 5 min.

Later, it was decreased to 60% in 5 min, to 100% in 5 min and remained constant during 2 min. Finally, it increased to 95% in 2 min and remained constant during 7 min. The total running time was 39 min. The injection volume was 10 μL.

The LC system was coupled to a hybrid mass spectrometer Q-Orbitrap Thermo Fisher Scientific (Q-Exactive^{TM}, Thermo Fisher Scientific, Bremen, Germany) using electrospray ionization (ESI) (HESI-II, Thermo Fisher Scientific, San Jose, CA, USA) in positive and negative ion mode. ESI parameters were as follows: spray voltage, 4 kV; sheath gas (N_2, 95%), 35 (arbitrary units); auxiliary gas (N_2, 95%), 10 (arbitrary units); S-lens RF level, 50 (arbitrary units); heater temperature, 305 °C, and capillary temperature, 300 °C. The mass spectra were acquired employing four alternating acquisition functions: (1) full MS, ESI+, without fragmentation (the higher collisional dissociation (HCD) collision cell was switched off), mass resolving power = 70,000 Full Width at Half Maximum (FWHM); AGC target = 1e6, scan time = 250 ms; (2) full MS, ESI-, without fragmentation (the HCD was switched off), mass resolving power = 70,000 Full Width at Half Maximum (FWHM); AGC target = 1e6, scan time = 250 ms; (3) data independent analysis (DIA), ESI+, setting HCD on, and collision energy = 30 eV, mass resolving power = 35,000 FWHM, scan time = 125 ms; (4) DIA, ESI- (setting HCD on, and collision energy = 30 eV), mass resolving power = 35,000 FWHM, scan time = 125 ms. The mass range in the full scan MS experiments was set to *m/z* 50-750. LC chromatograms were processed using Xcalibur^{TM} version 3.0, with Qualbrowser and Trace Finder 4.0 (Thermo Fisher Scientific, Les Ulis, France). Unknown analysis was carried out with Compound Discoverer^{TM} version 2.1 (Thermo Scientific, Les Ulis, France).

6. Total carotenoids

Fresh leaves (200 mg) were minced, and 1 mL KOH 60% w/w was added before sonication (10 min). The mixture was then heated at 40 ºC for 40 min, shaken, and kept in the dark under an inert atmosphere (nitrogen) at 4 ºC for 24 h. The mixture was then sonicated again (20 min), centrifuged at 2,000 g for 5 min, and then carotenoids were extracted with diethyl ether (1 mL). The extraction with diethyl ether was repeated twice and the extracts were put together for solvent evaporation under a nitrogen stream. The residue was dissolved in 5 mL acetone and the absorbance of this solution was measured at 444 nm in a 1-cm glass cuvette. Total carotenoids were quantified using a calibration

curve with a β-carotene standard in acetone (0-20 ppm) and results were reported as mg carotenoids/100 g fm.

7. Vitamin C

Extraction and quantification of vitamin C (ascorbic plus dehydroascorbic acids) was carried out according to Volden *et al*., (2009) with minor modifications. Fresh leaves were minced, and 1 g was collected and extracted with 20 mL of an aqueous solution of oxalic acid (1% m/v). The mixture was centrifuged at 2,500 g for 10 min, and then filtered to collect the filtrate (extract). The extract was filtered again through a 0.22 μm Millipore filter and 1 mL was used for chromatographic analysis. HPLC analysis of vitamin C was carried out in a Finnigan Surveyor chromatograph equipped with a diode-array detector (DAD) and a reverse-phase C18 column (HypersilGold, 250 mm × 4.6 mm i.d., 5 μm particle size) (Thermo Electron, Cambridge, UK). The mobile phase consisted of (A) methanol and (B) water with 0.1% oxalic acid in an isocratic mode 5% A and 95% B at 0.4 mL/min for 15 min. Detection was performed at 254 nm, and the injection volume was 10 μL. Ascorbic acid was quantified by external calibration and results were recorded as mg ascorbic acid/100 g fm. Determinations were carried out in triplicate and all data are reported as mean value ± standard deviation.

8. Antioxidant activity

Methanol extraction was carried out as described for total phenols and flavonoids analysis using a methanol:water mix (60:40 v/v). The antioxidant activity using the ABTS method was determined using a mother solution of $ABTS^{\bullet+}$ radical (2.2′-azinobis (3-ethylbenzothiazoline-6-sulfonic acid) in methanol (7 mM) and another one was determined to contain potassium persulfate (2.45 mM) in pH 7.0 phosphate buffer solution. The mixture was reacted at a 1:1 ratio at 25 °C in the absence of light for 16 h; subsequently, a 1950 μL of aliquot was removed from the $ABTS^{\bullet+}$ solution and 50 μL of the methanolic extract was added, mixed at 2000 rpm for 1 min in a vortex, and placed in darkness for 7 min at 25 °C (Re *et al*., 1999). The absorbance was measured at 734 nm. The DPPH method used the methodology described by Skenderidis *et al*., (2018) with some modifications. A $DPPH^{\bullet}$ stock solution (2.2-diphenyl-1-picrylhydrazyl) was prepared in methanol (0.25 mM) and agitated in an ultrasonic bath. From the $DPPH^{\bullet}$ stock solution, a working solution was prepared at 0.25 mM. From this solution, 1950 μL were collected to which 50 μL of the methanolic extract were added. The resulting 2000 μL

solution was vortexed at 2000 rpm for 30 sec and allowed to react in the dark at room temperature for 30 min. Then, the absorbance of the solution was read at 517 nm. The values of ABTS and DPPH were expressed as Trolox equivalent mg/100 g dry mass (mg TE/100 g dm).

9. MTT assays

The antiproliferative activity of IPL extracts on HT-29 human colon cancer cells and normal human fibroblast colon tissue CCD-18 cells was assayed as described by Lyashenko *et al*. (2021). Cultures were supplied by the Technical Instrumentation Service of the University of Granada (Granada, Spain). First, they were checked for the absence of *Mycoplasma* and bacteria. Then, cells were grown at 37 °C and 5% CO_2 humidified atmosphere in medium RPMI-1640 supplemented with 5% fetal bovine serum, 2 mM L-Glutamine, 1 mM sodium pyruvate, 0.125 mg/mL amphotericin, and 100 mg/mL penicillin-streptomycin.

All cultures were plated in 25 cm^2 plastic tissue culture flasks (Sarstedt, Newton, NC, USA). Cell culture and cell assay, that is, the MTT test, were accomplished as described by Lyashenko *et al*. (2021).

In the MTT assay, cells were divided into 96-well microtiter plates, adjusted to 1 × 104 cells/well, and cultivated in a medium at 37 °C and 5% CO_2 prior to adding the different extracts dissolved in the medium. The phenolic-containing extracts were supplied to cells dissolved in a mixture of methanol:water (60:40, v/v) and then in the culture medium at designed concentrations (0–1000 µg/mL). After 48 and 72 h of cell exposure, 5 mg/mL of an MTT solution was added to the culture medium to determine the viability of cells. The absorbance was recorded at 570 nm on an enzyme-linked immunosorbent assay (ELISA) plate reader (Thermo Electron Corporation, Sant Cugat del Valles, Barcelona, Spain). The formazan crystals produced were solubilized using 100 µL of DMSO. Cells without phenolic extracts were considered negative controls, which were used for all concentrations and tested extracts. Cell survival in exposed cultures relative to unexposed cultures was calculated, and the number of viable cells was calculated using the following equation:

Percentage of viable cells (%) = (Absorbance of treated cells/Absorbance of untreated cells) × 100%.

The concentrations causing 50% cell growth inhibition (GI_{50}) were calculated from the growth curves. Doxorubicin (98.0–102%, D1515) from Sigma-Aldrich (Madrid, Spain)

was used as a positive control, while DMSO and methanol were used as the negative (vehicle) controls. Phenolic extracts and controls were evaluated in three independent assays. Values presented are mean ± standard error of the mean. The SI of each extract was calculated as GI_{50} of the extract against the CCD-18 normal cell line/GI_{50} of the same extract against the HT-29 cancer cell line (Vichitsakul, 2023).

References

Ferrón-Carrillo, F.; Guil-Guerrero, J.L.; González-Fernández, M.J.; Lyashenko, S.; Battafarano, F.; Cunha-Chiamolera, T.P.L.; Urrestarazu, M. LED enhances plant performance and both carotenoids and nitrates profiles in lettuce. *Plant Foods Hum Nutr* 2021, *76*, 210-218.

Lyashenko, S.; González-Fernández, M.J.; Gómez-Mercado, F.; Yunusova, S.; Denisenko, O.; Guil-Guerrero, J.L. *Ribes* taxa: A promising source of γ-linolenic acid-rich functional oils. *Food Chem* 2019, *301*, 125309.

Lyashenko, S.; Fabrikov, D.; González-Fernández, M.J.; Gómez-Mercado, F.; López-Ruiz, R.; Fedorov, A.; De Bélair, G.; Urrestarazu, M.; Rodríguez-García, I.; Álvarez-Corral, M.; Guil-Guerrero, J.L. Phenolic composition and in vitro antiproliferative activity of Borago spp. seed extracts on HT-29 cancer cells. *Food Biosci* 2021, *42*, 101043.

Nájera, C.; Urrestarazu, M. Effect of the intensity and spectral quality of LED light on yield and nitrate accumulation in vegetables. *HortScience* 2019, 54, 1745–1750.

Peçanha, D.A.; Cunha-Chiamolera, T.P.L.; Chourak, Y.; Martínez-Rivera, E.Y.; Urrestarazu, M. Effect of the matric potential on growth and water, nitrate and potassium absorption of vegetables under soilless culture. *J Soil Sci Plant Nutr* 2021, *21*, 3493–3501.

Re, R.; Pellegrini, N.; Proteggente, A.; Pannala, A.; Yang, M.; Rice-Evans, C. Antioxidant activity applying an improved ABTS radical cation decolorization assay. *Free Rad Biol Med* 1999, *26*, 1231-1237.

Rodríguez, D.; Reca, J.; Martínez, J.; Lao, M.T.; Urrestarazu, M. Effect of controlling the leaching fraction on the fertigation and production of a tomato crop under soilless culture. *Sci Hortic* 2014, *179*, 153–157.

Singleton, V.L.; Orthofer, R.; Lamuela-Raventós, M.L. Analysis of total phenols and other oxidation substrates and antioxidants by means of folin-ciocalteu reagent. *Meth Enzymol* 1999, *299*, 152-178.

Skenderidis, P.; Kerasioti, E.; Karkanta, E.; Stagos, D.; Kouretas, D.; Petrotos, K.; Hadjichristodoulou, C.; Tsakalof, A. Assessment of the antioxidant and antimutagenic activity of extracts from goji berry of Greek cultivation. *Toxicol Rep* 2018, *5*, 251-257.

Urrestarazu, M.; Salas, M.C.; Valera, D.; Gómez, A.; Mazuela, P.C. Effects of heating nutrient solution on water and mineral uptake and early yield of two cucurbits under soilless culture. *J Plant Nutr* 2008, *31*, 527-553.

Vichitsakul, K.; Laowichuwakonnukul, K.; Soontornworajit, B.; Poomipark, N.; Itharat, A.; Rotkrua, P. Anti-proliferation and induction of mitochondria-mediated apoptosis by *Garcinia hanburyi* resin in colorectal cancer cells. *Heliyon* 2023, *22*, e16411.

Volden, J.; Bengtsson, G.B.; Wicklund, T. Glucosinolates, L-ascorbic acid, total phenols, anthocyanins, antioxidant capacities and colour in cauliflower (*Brassica oleracea* L. subsp. botrytis); effects of long-term freezer storage. *Food Chem* 2009, *112*, 967-976.

Zou, Y.; Lu, Y.; Wei, D. Antioxidant activity of a flavonoid-rich extract of *Hypericum perforatum* L. in vitro. *J Agric Food Chem* 2004, *52*, 5032-5039.
