# Supplementary material for: Growth parameters, phytochemicals, and antitumor activity of wild and cultivated ice plants (Mesembryanthemum crystallinum L.)
Source: Food Sci Nutr. 2024 Jun 21;12(9):6548–62. doi: 10.1002/fsn3.4286 (PMC11561852; doi:10.1002/fsn3.4286)
Supplement: Supplementary file 6 — Table S1 [file FSN3-12-6548-s003.pdf]

Supplementary Table 1. Composition of the nutrient solutions used for culturing *M. crystallinum* plants[†]

Electrical conductivity (dS m^{-1})	pH	Macronutrients (mM)						Micronutrients (μM)					
		NO_3^-	$H_2PO_4^-$	SO_4^{2-}	K^+	Ca^{2+}	Mg^{2+}	Fe	Mn	Cu	Zn	B	Mo
3.0	5.8	15.37	2.26	2.63	7.14	7.50	2.27	15	10	0.75	5	30	0.5
4.0	5.8	20.49	3.02	3.51	9.54	10.0	3.03	15	10	0.75	5	30	0.5
6.0	5.8	30.73	4.54	5.27	14.34	15.0	4.55	15	10	0.75	5	30	0.5

[†] Based on Sonneveld, C., Straver, N. (1994). Nutrient solutions for vegetables and flowers grown in water or substrates. *Series Voedingsoplossing Glastuinbouw*, *8*, 45.
