# Supplementary material for: Growth parameters, phytochemicals, and antitumor activity of wild and cultivated ice plants (Mesembryanthemum crystallinum L.)
Source: Food Sci Nutr. 2024 Jun 21;12(9):6548–62. doi: 10.1002/fsn3.4286 (PMC11561852; doi:10.1002/fsn3.4286)
Supplement: Supplementary file 7 — Table S2 [file FSN3-12-6548-s004.pdf]

Supplementary Table 2. Phenolic compounds identified in hydroalcoholic extracts (MeOH:H_2O, 60:40 v/v) of *M. crystallinum* leaves

Phenolic compound	Retention time (min)	Formula	Adduct	ESI mode	Expected m/z	m/z (Delta) (ppm)
4-*p*-coumaroylquinic acid	1.04	$C_{16}H_{18}O_8$	M-H	Negative	337.09179	-2.05263
Gallocatechin (-)	1.43	$C_{15}H_{14}O_7$	M+H	Positive	307.08123	5.29249
DL-*p*-hydroxyphenyllactic acid	6.41	$C_9H_{10}O_4$	M-H	Negative	181.05063	-2.70432
p-coumaric acid glucoside	6.53	$C_{15}H_{18}O_8$	M-H	Negative	325,09289	1.78596
Caffeic acid	6.91	$C_9H_8O_4$	M-H	Negative	179.03498	-3.44741
Sinapic acid	7.39	$C_{11}H_{12}O_5$	M-H	Negative	223.0612	-1.35466
Epicatechin gallate (-)	7.57	$C_{22}H_{18}O_{10}$	M-H	Negative	441.08272	-3.77551
p-Coumaric acid	7.77	$C_9H_8O_3$	M-H	Negative	163.04007	-4.58845
Quercetin	7.82	$C_{15}H_{10}O_7$	M-H	Negative	301.03538	-7.43404
Ferulic acid	7.95	$C_{10}H_{10}$O4	M-H	Negative	193.05063	-2.61526
Gallic acid	8.10	$C_7H_6O_5$	M-H	Negative	169.01425	-3.60113
4-Hydroxybenzoic acid/salicylic acid (isomers)	8.38	$C_8H_8O_4$	M-H	Negative	137.02442	-4.60859
2-Hydroxy-4-methoxybenzoic acid/vanillic acid (isomers)	8.94	$C_8H_8O_4$	M-H	Negative	167.03498	-4.69994
