# Supplementary material for: Growth parameters, phytochemicals, and antitumor activity of wild and cultivated ice plants (Mesembryanthemum crystallinum L.)
Source: Food Sci Nutr. 2024 Jun 21;12(9):6548–62. doi: 10.1002/fsn3.4286 (PMC11561852; doi:10.1002/fsn3.4286)
Supplement: Supplementary file 8 — Data S1 [file FSN3-12-6548-s002.docx]

**Supplementary Figures captions**

**Supplementary Figure 1.** Spectrum profiles of each LED lamp used as a treatment during the rooting and growing phase of M. crystallinum plants. L1: L18 T8 Roblan®; L2: L18 NS12 Valoya®; L3: L18 NS1 Valoya®; L4: L18 AP67 Valoya®.

**Supplementary Figure 2**. Electrical conductivity (A and B) and pH (C and D) of drainage versus different EC of the nutrient solutions (C1: 3.0; C2: 4.0; C3: 6.0 dS m^-1^) for *M. crystallinum* soilless culture. Different letters indicate significant differences (P<0.05) according to Tukey's test.

**Supplementary Figure 3**. Electrical conductivity (A and B) and pH (C and D) of drainage versus spectrum of illumination system for *M. crystallinum* soilless culture. L1: T8 Roblan®; L2: NS12 Valoya®; L3: L18 NS1 Valoya®; L4: L18 AP67 Valoya®. Different letters indicate significant differences (P<0.05) according to Tukey’s test.

**Supplementary Figure 4.** Dry mass (g plant ^-1^) versus different electrical conductivities of the nutrient solutions for *M. crystallinum* soilless culture (experimental results obtained in our facilities). Different letters indicate significant differences for each parameter (P<0.05) according to Tukey's test.
